# Preventive antibiotic therapy in acute stroke patients: A systematic review and meta-analysis of individual patient data of randomized controlled trials

**DOI:** 10.1177/23969873211056445

**Published:** 2021-11-03

**Authors:** Willeke F Westendorp, Jan-Dirk Vermeij, Craig J Smith, Amit K Kishore, John Hodsoll, Lalit Kalra, Andreas Meisel, Angel Chamorro, Jason J Chang, Yousef Rezaei, Mohammad R Amiri-Nikpour, Fabrizio A DeFalco, Jeffrey A Switzer, David J Blacker, Marcel GW Dijkgraaf, Paul J Nederkoorn, Diederik van de Beek

**Affiliations:** 1 Department of Neurology, Amsterdam Neuroscience, 26066 Amsterdam University Medical Center, Amsterdam, The Netherlands; 2 Department of Neurology, Sint Franciscusziekenhuis, Heusden-Zolder, Belgium; 3 Manchester Centre for Clinical Neurosciences, Geoffrey Jefferson Brain Research Centre, Manchester Academic Health Science Centre, Salford Royal NHS Foundation Trust, Salford, UK; 4 Division of Cardiovascular Sciences, Lydia Becker Institute of Immunology and Inflammation, 5292 University of Manchester, Manchester, UK; 5 Biostatistics Department, NIHR Biomedical Research Centre for Mental Health and Institute of Psychiatry, Psychology and Neurosciences, 4616 King’s College London, London, UK; 6 Clinical Neurosciences, 4616 King’s College Hospital NHS Foundation Trust, London, UK; 7 14903 Charité – Universitätsmedizin Berlin, Corporate Member of Freie Universität Berlin and Humboldt-Universität zu Berlin, Department of Neurology with Experimental Neurology, NeuroCure Clinical Research Center, Center for Stroke Research Berlin, Berlin, Germany; 8 Comprehensive Stroke Center, Department of Neuroscience, Hospital Clinic, 400745 University of Barcelona and August Pi I Sunyer Biomedical Research Institute (IDIBAPS), Barcelona, Spain; 9 Department of Critical Care Medicine, MedStar Washington Hospital Center, Washington, DC, USA; 10 Heart Valve Disease Research Center, Rajaie Cardiovascular Medical and Research Center, Iran University of Medical Sciences, Tehran, Iran; Department of Cardiology, Seyyed-al-Shohada Heart Center, Urmia University of Medical Science, West Azerbaijan, Iran; 11 Department of Neurology, Urmia University of Medical Sciences, West Azerbaijan, Iran; 12 Neurology Service, Villa dei Fiori Hospital – Acerra, Naples, Italy; 13 Department of Neurology, Medical College of Georgia, Augusta, ME, USA; 14 Perron Institute for Neurological and Translational Science, Nedlands, WA, Australia; Department of Neurology, 5728 Sir Charles Gairdner Hospital, Nedlands, WA, Australia; School of Medicine and Pharmacology, University of Western Australia; 15 Department of Epidemiology and Data Science, Amsterdam UMC, 26066 University of Amsterdam, Amsterdam, The Netherlands

**Keywords:** stroke, infection, antibiotic therapy

## Abstract

**Introduction:**

Infection after stroke is associated with unfavorable outcome. Randomized controlled studies did not show benefit of preventive antibiotics in stroke but lacked power for subgroup analyses. Aim of this study is to assess whether preventive antibiotic therapy after stroke improves functional outcome for specific patient groups in an individual patient data meta-analysis.

**Patients and methods:**

We searched MEDLINE (1946–7 May 2021), Embase (1947–7 May 2021), CENTRAL (17th September 2021), trial registries, cross-checked references and contacted researchers for randomized controlled trials of preventive antibiotic therapy versus placebo or standard care in ischemic or hemorrhagic stroke patients. Meta-analysis was performed by a one-step and two-step approach. Primary outcome was functional outcome adjusted for age and stroke severity. Secondary outcomes were infections and mortality.

**Results:**

4197 patients from nine trials were included. Preventive antibiotic therapy was not associated with a shift in functional outcome (mRS) at 3 months (OR1.13, 95%CI 0.98–1.31) or unfavorable functional outcome (mRS 3–6) (OR0.85, 95%CI 0.60–1.19). Preventive antibiotics did not improve functional outcome in pre-defined subgroups (age, stroke severity, timing and type of antibiotic therapy, pneumonia prediction scores, dysphagia, type of stroke, and type of trial). Preventive antibiotics reduced infections (276/2066 (13.4%) in the preventive antibiotic group vs. 417/2059 (20.3%) in the control group, OR 0.60, 95% CI 0.51–0.71, *p* < 0.001), but not pneumonia (191/2066 (9.2%) in the preventive antibiotic group vs. 205/2061 (9.9%) in the control group (OR 0.92 (0.75–1.14), *p* = 0.450).

**Discussion and conclusion:**

Preventive antibiotic therapy did not benefit any subgroup of patients with acute stroke and currently cannot be recommended.

## Introduction

Stroke is an important cause of death, accounting for 11.8% of deaths worldwide, and is the third most common cause of disability.^1^ Infections occur frequently after stroke and have been associated with unfavorable disease outcome.^2^ Several randomized clinical trials have investigated antibiotics to prevent infections after stroke.^3–8^ In a Cochrane systematic review, preventive antibiotic therapy compared to placebo or standard care did not reduce mortality or unfavorable outcome after stroke.^9^ Preventive antibiotics do reduce the number of infections after stroke and it could well be that some patients still benefit but not others, and how to select patients who could benefit is unclear. Our aim was to address this question with a meta-analysis of data from all trials for which individual patient data were available.

## Materials and methods

### Study selection

**Figure 1.**
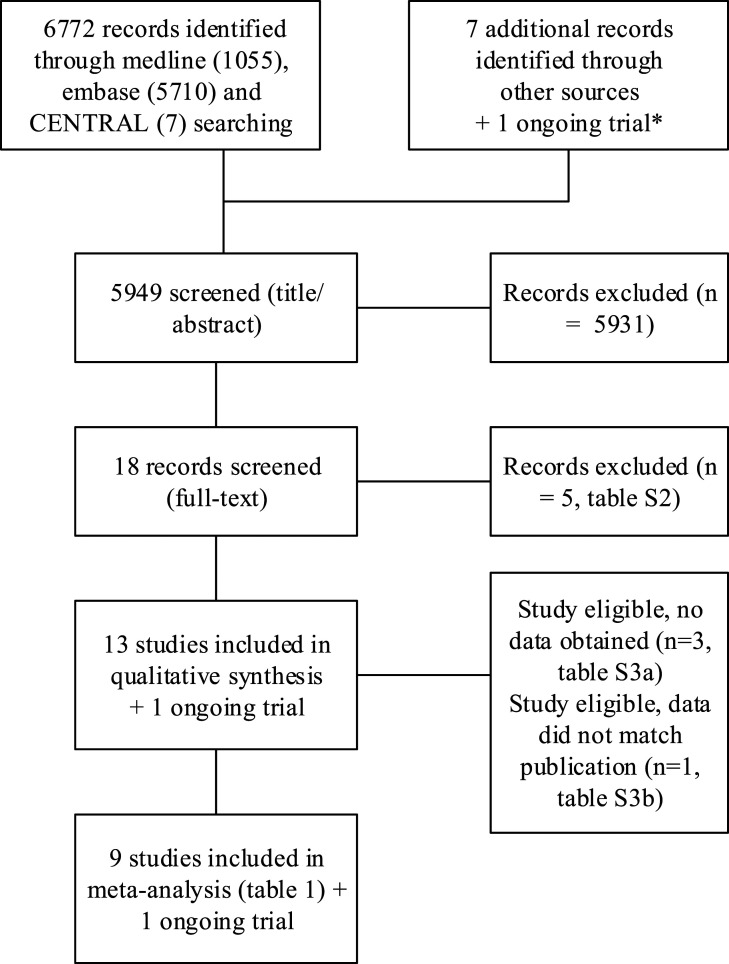
PRISMA flow-chart of the search.* Ongoing trial: www.precious-trial.eu. Search date: 7 May 2021.

A systematic literature review was undertaken in accordance with Centre for Reviews and Dissemination and Cochrane Collaboration guidance (Higgins, 2009; Figure 1, Supplementary figure 1). Search strategies are shown in Online only Supplementary table 1. Searches were undertaken in multiple electronic databases using pre-defined search criteria and terms. No language restrictions were applied to the search. We searched Ovid MEDLINE, Embase (1946–2020) and CENTRAL (17th September 2021), for randomized clinical trials of preventive antibiotic therapy in stroke. We cross-checked references, contacted researchers in the field, and principal investigators of included clinical trials to identify any other or unpublished material. We searched trial and research registers to identify ongoing studies (ClinicalTrials.gov (www.clinicaltrials.gov); ISRCTN Registry (www.isrctn.com); Stroke Trials Registry (www.strokecenter.org/trials); and WHO Registry Platform (apps.who.int/trialsearch). Two reviewers (WFW, JDV) screened the titles, abstract, and fulltext of the articles for inclusion. Possible disagreements were resolved in discussion with a third study reviewer (PN). After defining eligible studies, we contacted the principal investigators and co-authors of the trials for the individual patient data.

We included randomized controlled trials of adult (age 18 years or older) stroke patients (ischemic, intracerebral hemorrhage (ICH), or both) that randomized between preventive antibiotic therapy (route of administration: oral/by nasogastric tube, systemic, or intramuscular) and placebo or a standard care treatment that reported at least one of the following: infection or pneumonia rate, mortality, functional outcome. We excluded studies on patients with subarachnoid hemorrhage and studies with exclusively intubated and mechanically ventilated patients.

### Quality assessment

Study quality was assessed by two independent observers who had no role in the conduct of the included studies (AK, CS). The Cochrane Collaboration Tool was used for the assessment of study quality. The following items were evaluated: random sequence generation, (selection bias), allocation concealment (selection bias), blinding of participants and personnel (performance bias), blinding of outcome assessment (detection bias), incomplete outcome data (attrition bias), selective reporting (reporting bias), and other sources of bias.

### Definitions and outcome measurements

Data extraction and data definitions of variables for each trial are shown in Supplementary table 4 and 5.

General information was extracted for each trial: name of the author, year, and country of the study, type of stroke patients, number of patients included, whether placebo treatment or standard care was used as control group. For the individual patient analysis, we requested the following information from each trial (for each individual patient): sex; age; medical history (atrial fibrillation; chronic obstructive pulmonary disease (COPD); diabetes; baseline, pre-stroke disability (modified Rankin Scale [mRS])); stroke type; stroke severity (NIHSS); treatment with intravenous thrombolysis; use of urinary catheter; dysphagia; diagnosis of infection (pneumonia, urinary tract infection [UTI], other); time to diagnosis of infection; name, dosage, and frequency of preventive antibiotic therapy; time to first administration of preventive antibiotic therapy; functional outcome (mRS, Barthel Index [BI]) and mortality at discharge and at 3 months. Definition of variables across trials was investigated by completion of a questionnaire by the contact author of each trial. Data obtained from each trial were cross checked with the original publication.

Primary outcome was defined as functional improvement on the total range of the mRS. A secondary analysis of primary outcome was defined as the proportion of patients with unfavorable functional outcome at 3 months assessed on the mRS (mRS 3–6). Secondary outcomes were death, infection (any), and pneumonia during the follow-up period from each study. All analyses were adjusted for age and stroke severity and performed in the intention to treat populations of the included trials. Sensitivity analyses were performed for trials aimed at improving outcome by reducing infections (type 1 trial) and trials of neuroprotection with the antibiotic minocycline (type 2 trial).

For each of the primary and secondary outcomes, we performed prespecified subgroup analyses (for all trials and type 1 and 2 trials separate): age (≥/< 65 and 80 years), sex (male or female), stroke severity (NIHSS ≥ 5 and 10), type of stroke (ischemic vs hemorrhagic), treatment with thrombolysis (yes vs no), stratified risk of pneumonia (high risk patients vs low risk patients for pneumonia as defined by the externally validated ISAN-score^10^ for all strokes and A^2^DS^2^ score^11^ for ischemic stroke), dysphagia (based on initial swallow screening test), time to treatment (0–3 h, 3–6 h, 6–12 h, 12–24 h, >24 h), antibiotic class (tetracyclines, cephalosporins, fluoroquinolones, penicillins), quality of the study (placebo-controlled or open-label) and whether treatment was administered according to protocol.

We collected the following adverse events on trial level (as the type and definition of adverse events differed largely by trial): neurological (CT confirmed stroke extension, hemorrhagic stroke, recurrent stroke), general (cardiac, pulmonary, gastrointestinal events), development of antibiotic resistance (infections or colonization with resistant micro-organisms), and side-effects of medication (allergic reaction, diarrhea by *Clostridium difficile*, raised liver/plasma enzymes).

### Statistical analysis

For ordinal analyses of the mRS, the proportional odds assumption was not met, for example, the odds ratio (OR) for one level and the next was not constant and could not be summarized with a common OR. Therefore, analysis was performed using a two-step approach with an assumption-free ordinal analysis: Agresti’s generalized ORs (R statistical software, GenOdds package). This method calculates the odds that, if a pair of observations are randomly selected from two groups (preventive antibiotic therapy or control) the outcome in one group is higher than the other. This method has the additional advantage that it takes tied observations (“ties”) into consideration: ties are observations that belong to the same group of the ordinal variable (mRS) and therefore none of the two is higher than the other. Since ignoring tied observations consistently overestimates treatment effect compared to splitting tied observations, we split tied observations.^12,13^ The ordinal overall, and subgroup analysis followed a two-step approach: the assumption-free ordinal analysis, stratified by age and NIHSS (in categories), was performed on individual trial level, followed by a random-effect inverse variance pooling across trials.

Meta-analysis of dichotomous outcomes (unfavorable outcome, death, infection, pneumonia rate) was performed using a one-step approach; data from all studies were pooled while accounting for the clustering of patients within trials^14^). Logistic regression was used with “trial” and “trial*treatment” terms with adjustment for age and NIHSS (on a continuous scale). For subgroup analyses of dichotomous outcomes, the interaction term “treatment*pre-specified variable” and the prespecified variable separately were added to the model to test for statistically significant differences in treatment effects across the subgroups. SPSS version 26 and R statistical software were used for the analyses.

We assessed the amount of missing data for each outcome. Corresponding authors were contacted first for missing data. In case a certain variable was not collected in a trial, but was necessary for the analysis, this trial was excluded from the analysis. For each analysis, we describe the number of patients and trials on which this analysis was based. In case a variable was collected, but missing data occurred, the proportion of missing data was estimated. When this exceeded 5%, we analyzed whether data were missing at random or not, in case data were not missing at random the study was excluded from analysis. No data were imputed for this meta-analysis.

### Role of the funding source

The study sponsors had no role in the study design, collection, analysis, and interpretation of the data, or the decision to submit the manuscript for publication. W.F. Westendorp had full access to all data in the study. All authors approved and were responsible for submission of the manuscript.

## Results

In total, 18 publications were identified of which five were excluded (2 were not a randomized study, in 1 the randomization procedure was unclear, 1 study only included patients with indwelling catheters and in 1 study treatment with preventive antibiotic therapy was guided by procalcitonin levels; Supplementary table 2). Investigators of 13 trials were approached and 10 authors shared their data (2 studies were eligible for inclusion but we received no response from authors,^6,15^ 1 study was eligible but an author responded that the database was no longer available^16^; Supplementary table 3a). One additional trial was excluded because the received data did not match the original publication, leaving nine trials and 4197 stroke patients for the analysis (Table 1). Four trials were included as type 1 trials, aimed at improving outcome by reducing infections. These four trials included 3970 of 4197 (95%) of evaluated patients. Five trials were smaller type 2 trials, aimed at neuroprotection with the antibiotic minocycline. Risk of bias was generally low in type 1 studies and was moderate in some of the type 2 studies (Supplementary table 8).

**Table 1.** Included studies.

Author, year	Study population	Country	No. of patients	Antibiotic	Primary outcome	Secondary outcomes	Control group
Kalra et al., 2015^5^	Ischemic and hemorrhagic stroke patients with dysphagia	United Kingdom	1217	Local protocol	Post-stroke pneumonia in the first 14 days after stroke	Functional outcome (mRS) at 90 days, mortality, adverse events	Standard care
Westen-dorp et al., 2015^8^	Ischemic and hemorrhagic stroke patients	The Nether-lands	2538	Ceftriaxone	Functional outcome at 3 months on the mRS	Infections, pneumonia, mortality, adverse events	Standard care
Harms et al., 2008^4^	Patients with ischemic stroke (NIHSS>11) in middle cerebral artery territory	Germany	79	Moxifloxacin	Infection within 11 days after stroke	Neurological outcome (mRS), survival, immune-depression, induction of bacterial resistance	Placebo
Chamorro et al., 2005^3^	Ischemic and hemorrhagic stroke patients	Spain	136	Levo-floxacin	Infection within 7 days after stroke	Neurological outcome (mRS) and mortality at 90 days	Placebo
Chang et al., 2017^23^	Hemorrhagic stroke patients	USA	20	Minocycline	Adverse events	Change in serial NIHSS score, hematoma volume, MMP-9 measurements, 3-month functional outcome (mRS) and mortality	Placebo
Fouda et al., 2017^24^	Hemorrhagic stroke patients	USA	16	Minocycline	Serum concentrations of minocycline	ICH volume, inflammatory parameters	Standard care
Amiri-Nikpour et al., 2015^25^	Ischemic stroke patients	Iran	53	Minocycline	NIHSS at 3 months	NIHSS at 30, 60 days	Standard care
Kohler et al., 2013^26^	Ischemic and hemorrhagic stroke patients	Australia	92	Minocycline	Survival free of handicap (mRS ≤2) at day 90	NIHSS at day 7, Barthel index at 90 days	Standard care
Blacker et al., 2013^27^	Ischemic stroke patients that received thrombolysis	Australia	46	Minocycline	Hemorrhagic transformation on CT-scan	—	Standard care

The baseline characteristics were similar in antibiotic and control or placebo groups within the nine studies (Table 2). 2100 patients (50.0%) received preventive antibiotic treatment and 2097 (50.0%) standard care or placebo. Ischemic stroke was diagnosed in 3580 of 4196 patients (85%), hemorrhagic stroke in 467 patients (11%), TIA in 94 patients (2%), and another diagnosis was made in 55 patients (1%).

**Table 2.** Baseline characteristics of all patients.

Characteristic	Preventive antibiotic treatment (*n* = 2100)	Standard care/placebo (*n* = 2097)
Age (years)	75 (65–82)	75 (65–83)
Male sex	52 (1095/2097)	52 (1091/2096)
Medical history		
Obstructive pulmonary disease	9 (176/2055)	7 (149/2047)
Diabetes mellitus	20 (418/2098)	20 (420/2095)
Atrial fibrillation	22 (436/1995)	23 (463/1990)
Pre-stroke disability (mRS)	0 (0–1)	0 (0–1)
Stroke severity (NIHSS)	8 (4–15)	7 (4–15)
Stroke type		
Ischemic	85 (1781/2100)	86 (1799/2096)
Hemorrhagic	12 (252/2100)	10 (215/2096)
TIA	2 (44/2100)	2 (50/2096)
Other diagnosis	1 (23/2100)	2 (32/2096)
Intravenous thrombolysis	32 (659/2061)	31 (634/2057)
Dysphagia	51 (922/1793)	51 (918/1795)
Use of urinary cathether	20 (262/1314)	22 (286/1315)

Data in % (*n*/*N*), median with interquartile range or mean with standard deviation.

mRS: modified Rankin scale; NIHSS: National Institute of Stroke Severity Scale; TIA: transient ischemic attack.

Preventive antibiotic therapy was not associated with a shift in functional outcome on the mRS at 3 months (OR 1.13, 95%CI 0.98–1.31, *p* = 0.0896, moderate heterogeneity, I^2^ 47%, Supplementary figure 2 and 3, table 3). Preventive antibiotics was also not associated with favorable outcome in the analysis using dichotomization of the mRS (Table 3). However, in type 2 trials, preventive minocycline was associated with worse functional outcome on the total range of the mRS at 3 months (OR 1.46, 95%CI 1.02–2.09, *p* = 0.04, Supplementary figure 2).

**Table 3.** Study outcomes.

	Antibiotics (*n* = 2100) % (*n*/*N*)	Standard care/placebo (*n* = 2097) % (*n*/*N*)	Odds ratio (95%CI)	*p* value
Primary outcome				
Functional worsening on mRS	-	-	1.13 (0.98–1.31)	0.09
Type 1			1.08 (0.94–1.25)	0.27
Type 2			1.46 (1.02–2.09)	0.04
Unfavorable functional outcome^a^	52.0 (1057/2032)	52.2 (1060/2029)	0.85 (0.60–1.19)	0.348
Type 1	53.0 (1033/1949)	53.1 (1032/1942)	0.85 (0.60–1.19)	0.348
Type 2	28.9 (24/83)	32.2 (28/87)	0.25 (0.04–1.44)	0.120
Secondary outcomes				
Death	16.7 (344/2066)	15.3 (316/2067)	1.13 (0.95–1.36)	0.165
Type 1	17.3 (339/1957)	16.0 (313/1952)	1.13 (0.94–1.35)	0.199
Type 2	4.6 (5/109)	2.6 (3/115)	1.82 (0.36–9.12)	0.468
Any infection	13.4 (276/2066)	20.3 (417/2059)	0.60 (0.51–0.71)	0.001
Pneumonia	9.2 (191/2066)	9.9 (205/2061)	0.92 (0.75–1.14)	0.450
UTI	3.6 (74/2066)	9.7 (201/2062)	0.34 (0.24–0.48)	<0.001
Other infection	1.7 (35/2066)	1.9 (39/2062)	0.90 (0.56–1.46)	0.639

All analyses are adjusted for age and stroke severity (NIHSS).

mRS: modified Rankin Scale; UTI: urinary tract infection.

^a^Unfavorable functional outcome: mRS 3–6 or Barthel Index <60 or deceased.

Preventive antibiotic therapy was associated with a decrease of any infections (13.4% in the preventive antibiotic group vs 20.3% in the control group, OR 0.60, 95% CI 0.51–0.71, *p* < 0.001). This was mainly driven by a decrease in UTI (74 of 2066 [3.6%] in the preventive antibiotic group vs. 201 of 2062 [9.7%] in the control group, OR 0.34 95%CI 0.26–0.45, *p* < 0.001), while the proportion of patients with pneumonia and other infections were similar between groups (191 of 2066 [9.2%] in the preventive antibiotic group vs. 205 of 2061 [9.9%] in the control group, OR 0.92 (0.75–1.14), *p* = 0.450 and 1.7 35/2066 [1.7%] vs 39/2062 [1.9%], OR 0.90 (0.56–1.46), *p* = 0.639). Any infection (OR 2.65, 95% CI 2.08–3.37, *p* < 0.001) and pneumonia (OR 6.76 95% CI 4.34–10.54, *p* < 0.001) were associated with unfavorable functional outcome in analyses adjusted for age and stroke severity.

Sex, stroke severity, type of stroke, treatment with thrombolysis, subgroups based on risk scores ISAN and A^2^DS^2^ score, dysphagia, time to treatment, antibiotic class, placebo-controlled versus open-label study, and whether treatment was administered according to protocol or not did not significantly influence treatment response of preventive antibiotic therapy (Supplementary table 12–14).

**Figure 2.**
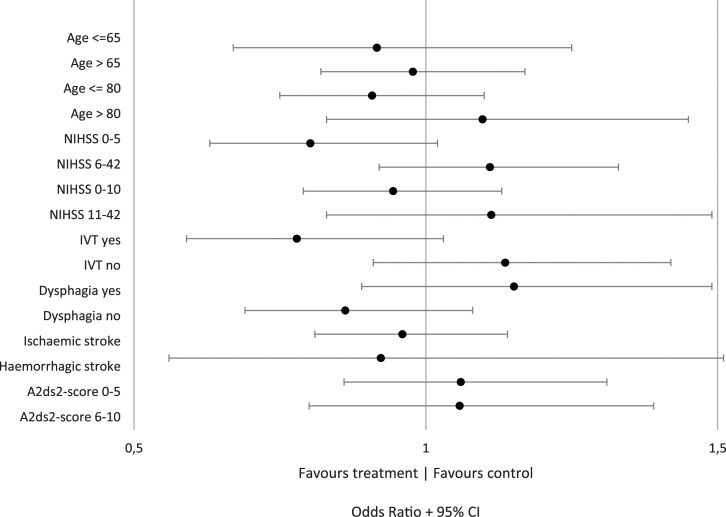
Subgroup analysis for unfavorable outcome (mRS 3–6) for all trials. This figure shows the odds ratios for unfavorable outcome in patients randomized to antibiotic therapy versus patients randomized to standard care, for each subgroup of patients (*y*-axis). An odds ratio larger than one favors control, smaller than one favors antibiotic therapy.

The analysis of preventive antibiotics in patients with lower stroke severity (NIHSS ≤5) suggested a favorable effect (*p*-value interaction 0.028) in type 1 trials (Figure 2). However, this analysis was merely a comparison between trials as 90% of patients with NIHSS ≤5 were derived from 1 trial and 1 other trial only included patients with NIHSS >5 (Supplementary table 19).

The analyses of longer time to treatment suggested possible harm (*p*-value interaction 0.02, Supplementary table 15). The analysis for those treated with iv-thrombolysis showed a trend toward benefit (301 of 639 [47%] with unfavorable outcome in the preventive antibiotic group vs. 327 of 607 [54%] in the control group, *p*-value interaction 0.08, Supplementary table 15). Overall, the number of adverse events was comparable in both treatment arms (Supplementary table 18). A post-hoc sample size analysis for primary outcome showed sufficient power to detect a clinical meaningful effect even in smaller subgroups of patients (Supplementary appendix page 28).

## Discussion

This individual patient meta-analysis did not show benefit of preventive antibiotic therapy in stroke patients. Preventive antibiotic therapy did reduce the occurrence of any infection but this was largely driven by a decrease in the proportion of patients with UTI but not a decrease in the incidence of pneumonia which was independently associated with unfavorable functional outcome. Extensive exploration of pre-specified subgroups did not show robust evidence of benefit in any particular subgroup. The suggested benefit for those treated with iv-thrombolysis is likely to have occurred by chance and the apparent harm in patients with longer time to treatment is likely to be due to confounding. The effect of preventive antibiotic therapy found in patients with lower stroke severity (NIHSS < 5) was merely a comparison between trials as 90% of patients with NIHSS < 5 were derived from 1 trial.

Preventive antibiotics did not reduce occurrence of pneumonia. In this meta-analysis, approximately 1 in 10 patients suffered from pneumonia, which is in line with previous evidence.^2^ Pneumonia is one of the most common complications after stroke and contributes strongly to unfavorable outcome.^17^ In a cohort study of 8251 stroke patients, the occurrence of pneumonia was associated with less favorable outcome at discharge (OR 0.2, 95% CI 0.14–0.29, *p*-value 0.001) and increased 1-year mortality (OR 3.0, 95%CI 2.5–3.7, *p*-value <0.0001).^18^ The lack of effect of antibiotic therapy on the incidence of pneumonia raises the question whether the type and timing of antibiotic therapy may have been important. The antibiotic therapies used in included trials cover most of the pathogens associated with pneumonia after stroke,^19^ although anaerobes might not have been covered in the PASS trial as ceftriaxone did not cover anaerobic pathogens. The preferred antibiotic regimen used in 70% of patients in the STROKE-INF trial did cover anaerobic pathogens but in this trial pneumonia frequency was also not reduced. Next, timing of start of antibiotic therapy might have been too late. In the two largest trials (PASS and STROKE-INF), patients had to start therapy within 24 and 48 h, respectively. This might have been too late as 75% of infections are diagnosed within 3 days after admission.^20^ The time to treatment subgroup analysis showed that delay in treatment was associated with unfavorable outcome in the analysis of all trials and type 2 trials, but not in the separate analysis of the type 1 trials that were specifically aimed at preventing infections. In addition, it is likely that confounding exists in this subgroup analysis as patients who present early to the hospital benefit more from more rapid specialist stroke unit care and we could not correct for this possible confounding. Another potential explanation for the lack of effect of preventive antibiotics on outcome is that pneumonia after stroke could also incorporate a non-infective respiratory syndrome which antibiotics cannot prevent.^21^ This respiratory syndrome may relate to chemical injury caused by inhalation of sterile gastric contents rather than a true infection by pathogenic bacteria, and therefore may not be preventable with antibiotic therapy.^21^ Because antibiotic therapy did not reduce pneumonia frequency, it remains unclear whether pneumonia has a causal relationship with unfavorable outcome or is merely an epi-phenomenon of severe stroke.

**Figure 3.**
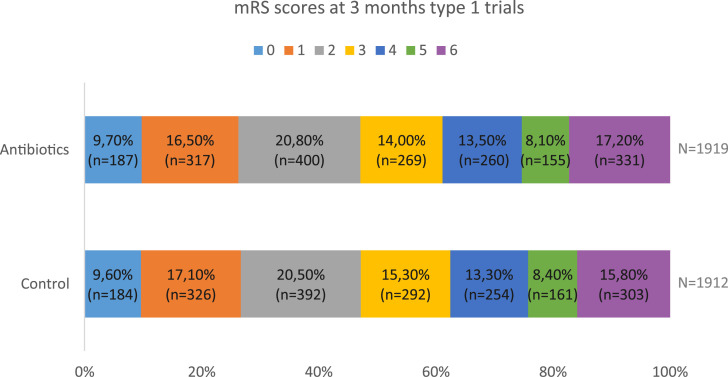
Modified Rankin Scale score at 3 months for patients included in type 1 trials.

In this analysis, we aimed to include all the available randomized trial evidence but some data remained unavailable despite our best efforts. For type 1 trials, it is unlikely that the one missing trial would change the results of this analysis, as the number of included patients in this analysis is high (3970) and the missing study only included 60 patients.^6^ In contrast, for type 2 trials focused on the neuroprotective effect of minocycline two missing trials could have impacted our results, in particular because the number of patients in these missing trials is similar to the number of patients included in the current analysis of type 2 trials.^15,16^ Indeed, in a recent study-level meta-analysis of minocycline trials that included these trials, a trend toward a favorable effect (mRS 0–2) of minocycline on functional outcome was seen (RR = 1.31; 95% CI 0.98–1.74, *p* = 0.06).^22^ In addition, risk of bias was moderate in some of the studies on minocycline. Therefore, the evidence on the effect of minocycline on outcome in stroke patients is inconclusive. As trials for minocycline were small and probably underpowered, a subsequent larger trial could give more reliable estimates on the efficacy of minocycline (Figure 3).

In the current analysis, we did not adjust for multiple comparisons. As we did not find a robust effect in one of the subgroups, this is unlikely to have influenced results.

We found no benefit of preventive antibiotics aimed at reducing infection in prespecified subgroups of patients. One ongoing trial might significantly change the abovementioned results: the PRECIOUS-trial (ISRCTN 82217627). In the PRECIOUS-trial, not only preventive antibiotic therapy to prevent infections (ceftriaxone) is investigated, but also two other pharmacological interventions for post-stroke complications: metoclopramide for aspiration and paracetamol for fever. As the sample size of the study is 3800 patients, this study has the potential to change the abovementioned results for patients aged 66 or older.

In conclusion, preventive antibiotic therapy in patients with acute stroke decreases any infections but does not reduce pneumonia or unfavorable functional outcome. There were no significant treatment effects in any of the pre-specified subgroups. Preventive antibiotics did not benefit all or any subgroup of patients with acute stroke and can currently not be recommended.

## Supplementary Material

Supplementary material
