# Supplementary material for: Preventive antibiotic therapy in acute stroke patients: A systematic review and meta-analysis of individual patient data of randomized controlled trials
Source: Eur Stroke J. 2021 Nov 3;6(4):385–94. doi: 10.1177/23969873211056445 (PMC8948510; doi:10.1177/23969873211056445)
Supplement: Supplementary material [file sj-pdf-1-eso-10.1177_23969873211056445.pdf]

Supplemental file

Preventive antibiotic therapy in subgroups of acute stroke patients: an individual patient meta-analysis

Contents **Page**

Table S1. Search strategy 2

Table S2. Excluded studies 4

Table S3. Studies eligible but no data obtained 4

Table S4. Variables extracted from each trial on individual patient level 5

Table S5. Data definitions across trials 6-9

Table S6. Baseline characteristics of type 1 trials 10

Table S7. Baseline characteristics of type 2 trials 11

Table S8. Risk of bias assessment 12

Table S9. Outcomes for each trial 13

Table S10. Unfavorable outcome (mRS 3-6) at 3 months in all patients per trial 14

Figure S1. Unfavorable outcome (mRS 3-6) at 3 months per trial 14

Table S11. Ordinal analysis mRS in all patients per trial 15

Figure S2. mRS scores at 3 months for all patients 16

Figure S3. mRS scores at 3 months for patients included in type 2 trials 16

Table S12. Subgroup analyses for the primary outcome of functional outcome on total range of mRS for all trials 17

Table S13. Subgroup analyses for the primary outcome of functional outcome on total range of mRS for type 1 trials 18

Table S14. Subgroup analyses for the primary outcome of functional outcome on total range of mRS for type 2 trials 19

Table S15. Subgroup analyses for the primary outcome of unfavorable functional outcome (mRS 3-6) for all trials. 20

Table S16. Subgroup analyses for the primary outcome of unfavorable functional outcome (mRS 3-6) for type 1 trials. 22

Table S17. Subgroup analyses for the primary outcome of unfavorable functional outcome (mRS 3-6) for type 2 trials. 24

Table S18. Adverse events 26

Table S19. Analysis of unfavorable outcome with NIHSS 5 cutoff 27

Post-hoc sample size analysis 28

Supplemental Table 1. Search Strategy		
**Ovid MEDLINE (1946 – May 7th 2020)**		
	**Searches**	**Results**
1	cerebrovascular disorders/ or exp basal ganglia cerebrovascular disease/ or exp brain ischemia/ or exp carotid artery diseases/ or exp cerebrovascular trauma/ or exp intracranial arterial diseases/ or exp intracranial arteriovenous malformations/ or exp "intracranial embolism and thrombosis"/ or exp intracranial hemorrhages/ or stroke/ or exp brain infarction/ or vasospasm, intracranial/ or vertebral artery dissection/	364994
2	(stroke$ or poststroke$ or cva$ or cerebrovascular$ or cerebral vascular).tw.	313781
3	((cerebral or cerebellar or brain$ or vertebrobasilar) adj5 (infarct$ or isch?emi$ or thrombo$ or apoplexy or emboli$)).tw.	94335
4	((cerebral or intracerebral or intracranial or brain or cerebellar or subarachnoid) adj5 (haemorrhage or hemorrhage or haematoma or hematoma or bleeding or aneurysm)).tw.	72576
5	1 or 2 or 3 or 4	558713
6	Antibiotic Prophylaxis/	14374
7	exp Anti-Bacterial Agents/	747215
8	(antibiotic$ or anti-bacterial or anti bacterial or antibacterial or bacteriocid$ or anti-mycobacterial or anti mycobacterial or antimycobacterial or anti-infect$ or anti infect$).tw.	427649
9	(amoxicillin or amphotericin b or ampicillin or calcimycin or cephalosporin$ or cephalothin or cephamycin$ or chloramphenicol or dactinomycin or doxycycline or erythromycin or fluoroquinolone$ or gentamicin$ or kanamycin or minocycline or neomycin or oxytetracycline or penicillin or streptomycin or tetracycline or vancomycin).tw.	270851
10	7 or 8 or 9	1007805
11	exp infection/ or exp bacterial infections/ or exp infection control/ or exp fever/ or exp inflammation/	2946884
12	(infection$ or sepsis or septicaemia or septicemia or pneumonia or bacteremia or bacteraemia or inflammation or fever or blood poisoning).tw.	2138905
13	11 or 12	3893655
14	(prophyla$ or prevent$ or premedicat$ or incidence or occurrence).tw.	2593240
15	prevention control.fs.	1336031
16	15 or 14	3433905
17	10 and 13 and 16	115044
18	6 or 17	120953
19	5 and 18	1055
**Embase classic + Embase (1947 – May 7th 2020)**		
**#**	**Searches**	**Results**
1	cerebrovascular disease/ or basal ganglion hemorrhage/ or exp brain hematoma/ or exp brain hemorrhage/ or exp brain infarction/ or exp brain ischemia/ or exp carotid artery disease/ or exp cerebral artery disease/ or cerebrovascular accident/ or exp cerebrovascular malformation/ or exp intracranial aneurysm/ or exp occlusive cerebrovascular disease/ or stroke/	782967
2	stroke unit/ or stroke patient/	37529
3	(stroke$ or poststroke$ or cva$ or cerebrovascular$ or cerebral vascular).tw.	507145
4	((cerebral or cerebellar or brain$ or vertebrobasilar) adj5 (infarct$ or isch?emi$ or thrombo$ or apoplexy or emboli$)).tw.	137661
5	((cerebral or intracerebral or intracranial or brain or cerebellar or subarachnoid) adj5 (haemorrhage or hemorrhage or haematoma or hematoma or bleeding or aneurysm)).tw.	109930
6	1 or 2 or 3 or 4 or 5	939203
7	antibiotic prophylaxis/	33976
8	exp antibiotic agent/	1681145
9	(antibiotic$ or anti-bacterial or anti bacterial or antibacterial or bacteriocid$ or anti-mycobacterial or anti mycobacterial or antimycobacterial or anti-infect$ or anti infect$).tw.	615316
10	(amoxicillin or amphotericin b or ampicillin or calcimycin or cephalosporin$ or cephalothin or cephamycin$ or chloramphenicol or dactinomycin or doxycycline or erythromycin or fluoroquinolone$ or gentamicin$ or kanamycin or minocycline or neomycin or oxytetracycline or penicillin or streptomycin or tetracycline or vancomycin).tw.	378306
11	8 or 9 or 10	1955352
12	exp infection/ or infection control/ or infection risk/ or fever/ or exp inflammation/	6504644

13	(infection$ or sepsis or septicaemia or septicemia or pneumonia or bacteremia or bacteraemia or inflammation or fever or blood poisoning).tw.	3012970
14	12 or 13	7087118
15	(prophyla$ or prevent$ or premedicat$ or incidence or occurrence).tw.	3699696
16	prophylaxis/	129564
17	16 or 15	3724061
18	11 and 14 and 17	198954
19	infection prevention/ or exp infection/pc	373699
20	11 and 19	73844
21	7 or 18 or 20	245223
22	6 and 21	5710
**Cochrane Central (search run on Fri Sep 17 2021)**		**Results**
	**Searches**	
1	((stroke$ or poststroke$ or cva$ or cerebrovascular* or cerebral vascular)):TI,AB,KY"	20732
2	(((cerebral or cerebellar or brain$ or vertebrobasilar) adj5 (infarct$ or isch?emi$ or thrombo$ or apoplexy or emboli$))):TI,AB,KY	142
3	(((cerebral or intracerebral or intracranial or brain or cerebellar or subarachnoid) adj5 (haemorrhage or hemorrhage or haematoma or hematoma or bleeding or aneurysm))):TI,AB,KY	9786
4	1 OR 2 OR 3	28984
5	((antibiotic$ or anti-bacterial or anti bacterial or antibacterial or bacteriocid$ or anti-mycobacterial or anti mycobacterial or antimycobacterial or anti-infect$ or anti infect$)):TI,AB,KY	13632
6	((amoxicillin or amphotericin b or ampicillin or calcimycin or cephalosporin$ or cephalothin or cephamycin$ or chloramphenicol or dactinomycin or doxycycline or erythromycin or fluoroquinolone$ or gentamicin$ or kanamycin or minocycline or neomycin or oxytetracycline or penicillin or streptomycin or tetracycline or vancomycin)):TI,AB,KY	20260
7	7 OR 5 OR 6	29471
8	((infection$ or sepsis or septicaemia or septicemia or pneumonia or bacteremia or bacteraemia or inflammation or fever or blood poisoning)):TI,AB,KY	83126
9	((prophyla$ or prevent$ or premedicat$ or incidence or occurrence)):TI,AB,KY"	132756
10	4 and 7 and 8 and 9	7

**Supplemental table 2. Excluded studies based on fulltext screening**	
**Author, year**	**Reason for exclusion**
Nyren et al, 1981 (23)	Solely patients with indwelling catheters
Maijkowski et al, 1982 (24)	Randomization procedure unclear
Fagan et al, 2010 (25)	Not a randomized study
Switzer et al, 2011 (26)	Not a randomized study
Ulm et al, 2017 (27)	Treatment with preventive antibiotic therapy was guided by procalcitonin levels

**Supplemental table 3a. Studies eligible for including but no data obtained**	
**Author, year**	**Reason for exclusion**
Padma Srivastava et al, 2012 (15)	Study was eligible for inclusion, no response from authors
Schwarz et al, 2007 (6)	Study was eligible for inclusion, no response from authors
Lampl et al, 2007 (16)	Study was eligible for inclusion, contact with author: database no longer available, all authors retired or working elsewhere
**Supplemental table 3b. Excluded studies based on discrepancies in data with original publication**	
DeFalco et al, 1998 (28)	Received data did not match original publication

Supplemental table 4. Variables extracted from each trial on individual patient level									
**Variable**	**Type 1 trial (aimed at prevention of infection)**				**Type 2 trial (Minocyclin)**				
	**Kalra et al, 2015 (5)**	**Westendorp et al, 2015 (8)**	**Harms et al, 2008 (4)**	**Chamorro et al, 2005 (3)**	**Chang et al, 2017 (29)**	**Fouda et al, 2017 (30)**	**Blacker et al, 2015 (31)**	**Amiri nikpour et al, 2015 (32)**	**Kohler et al, 2013 (33)**
Randomization	X	X	X	X	X	X	X	X	X
Sex	X	X	X	X	X	X	X	X	X
Age	X	X	X	X	X	X	X	X	X
Medical history of atrial fibrillation	X	X	X	-	X	-	X	-	X
Medical history of chronic obstructive pulmonary disease	X	X	X	X	-	-	X	-	X
Medical history of diabetes	X	X	X	X	X	X	X	X	X
Score on modified Rankin Scale before stroke	X	X	-	-	X	-	X	-	-
Stroke type	X	X	X	X	X	X	X	X	X
Stroke severity at admission on NIHSS	X	X	-	X	X	X	X	X	X
Treatment with iv-thrombolysis	X	X	-	X	X	X		X	X
Use of urinary catheter	-	X	-	-	X	-	-	-	-
Dysphagia	X	X	-	-	-	-	-	-	-
Diagnosis of infection during admission	X	X	X	X	X	-	X	-	X
Days to diagnosis of infection	-	X	X	X	X	-	-	-	X
Diagnosis of pneumonia during admission	X	X	X	X	X	-	X	-	X
Days to diagnosis of pneumonia	-	X	X	X	X	-	-	-	X
Diagnosis of UTI during admission	-	X	X	X	X	-	X	-	X
Days to diagnosis of UTI	-	X	X	X	X	-	-	-	X
Diagnosis of other infection during admission	-	X	-	X	X	-	X	-	X
Days to diagnosis of other infection	-	X	-	X	X	-	-	-	X
Length of hospital stay	X	X	-	-	X	-	-	-	-
Discharge - score on modified Rankin Scale	-	-	-	-	X	-	-	-	-
Discharge - unfavorable outcome	-	-	X		X	-	-	-	-
Discharge - mortality	-	X	X	-	X	-	X	X	X
3 months – score on modified Rankin Scale	X	X	-	X	X	X	X	-	X
3 months - NIHSS	-	-	X	X	X	X	-	X	-
3 months – unfavorable functional outcome *	X	X	-	X	X	X	X	-	X
3 months – barthel index	-	-	X	-	X	-	X	-	X
Therapy with preventive antibiotic therapy: name, class, route and dosage	-	X	X	X	X	X	X	X	X
Number of days of treatment with preventive antibiotic therapy	-	X	-	X	X	X	X	X	X
Treatment per protocol	X	X	X	X	X	X	X	X	X
Time to first dose of preventive antibiotic therapy	-	X	-	-	X	X	X	-	X
Serious adverse events	X	X					X		X
* mRS=3-6; BI <60 or deceased									

Supplemental table 5. Definition of baseline characteristics (when ascertained)	
Age	All trials: age of the patient in years at day of inclusion in the trial
Sex	All trials: sex of the patient as reported by patient
Medical history of atrial fibrillation	Kalra et al: -<br>Westendorp et al: assessed by physician who admitted the patient from medical records, new diagnosis if atrial fibrillation on ECG at admission.<br>Harms et al: taken from the medical records of the patient<br>Chang et al: by reported history or atrial fibrillation noted during hospitalization/ telemetry<br>Blacker et al: by standard ECG, or documentation in medical record<br>Kohler et al: by standard ECG, or documentation in medical record
Medical history of obstructive pulmonary disease	Kalra et al: -<br>Westendorp et al: assessed by physician who admitted the patient from medical records.<br>Harms et al: taken from the medical records of the patient<br>Chamorro et al: -<br>Blacker et al: documentation in medical record<br>Kohler et al: documentation in medical record
Medical history of diabetes mellitus	Kalra et al: -<br>Westendorp et al: assessed by physician who admitted the patient from medical records.<br>Harms et al: taken from the medical records of the patient<br>Chamorro et al: -<br>Chang et al: by reported history<br>Fouda et al: documented history in medical record or reported history by patient or family<br>Blacker et al: new diagnosis if BSL > 11, or documentation in medical record<br>Amiri-Nikpour et al: any drugs used for treating diabetes and medical documents confirming the diagnosis of diabetes. The patient and his/her family members/proxy were also used for confirming the diagnosis in condition it was found to be reliable. In addition, we also routinely checked fasting blood glucose and blood glucose to assess the diabetes status of patients.<br>Kohler et al: new diagnosis if BSL > 11, or documentation in medical record
Pre-stroke disability (mRS)	Kalra et al: -<br>Westendorp et al: assessed by physician at admission<br>Chang et al: assessed at admission by neurologist<br>Blacker et al: assessed at admission on modified rankin scale
Stroke type	Kalra et al: -<br>Westendorp et al: physician's assessment, always included head-CT-scan, some cases also MRI<br>Harms et al: clinical diagnosis of an acute ischemic stroke in the middle cerebral artery territory and NIHSS>11 between 9 and 36h of symptoms onset (exclusion of ICH by CT)<br>Chamorro et al: brain CT or MRI<br>Chang et al: study was only hemorrhagic stroke/ ICH. Defined by CTH.<br>Fouda et al: CT-scan<br>Blacker et al: CT or MRI<br>Amiri-Nikpour et al: CT-scan<br>Kohler et al: CT or MRI
Stroke severity	Kalra et al: assesses on NIHSS by physician<br>Westendorp et al: assessed on NIHSS by resident neurology / neurologist, in most, but not all, centers trained in NIHSS scoring<br>Harms et al: assessed on NIHSS by study physicians (neurologists trained in stroke medicine and NIHSS scoring)<br>Chamorro et al: scored on NIHSS by physician<br>Chang et al: neurologist (myself) on NIHSS; yes trained in NIHSS<br>Fouda et al: co-investigators trained for NIHSS scoring<br>Blacker et al: on NIHSS, usually a neurology trainee, sometimes a consultant neurologist; MOST (not all), had done NIHSS training. Clinicians all had access to written aids to calculate the NIHSS.<br>Amiri-Nikpour et al: assessed on NIHSS by physicians trained for NIHSS assessment<br>Kohler et al: on NIHSS, usually by a neurology trainee, sometimes a consultant neurologist; MOST (not all), had done NIHSS training. Clinicians all had access to written aids to

	calculate the NIHSS.
Intravenous thrombolysis	Kalra et al: -<br>Westendorp et al: Actilyse 1mg/ml according to the weight of a patient<br>Harms et al: Actilyse according to the guidelines (0,9mg/kg bw in max 90mg, 10% Bolus, 90% over 60min; within 3,5 hours)<br>Chamorro et al: no patients received iv-thrombolysis<br>Chang et al: not applicable (solely hemorrhagic stroke included)<br>Fouda et al: not applicable<br>Blacker et al: alteplase, 0.9mg/kg as per standard protocol based on state and national guidelines.<br>Amiri-Nikpour et al: no patients received iv-thrombolysis<br>Kohler et al: alteplase, 0.9mg/kg as per standard protocol based on state and national guidelines.
Use of urinary catheter	Westendorp et al: use of urinary catheter during (a part of) the admission<br>Chang et al: use of urinary catheter during admission
Dysphagia	Kalra et al: inclusion in the trial was based on dysphagia. Dysphagia-trained nursing staff assessed swallowing using the standard bedside swallowing assessment test consisting of measuring levels of consciousness, oromotor function, and consumption of water or food.<br>Westendorp et al: the presence of any type of dysphagia as assessed by a dysphagia trained nurse and / or speech therapist.
Diagnosis of infection / pneumonia / UTI / other infection and time-frame in which this was assessed.	Kalra et al: diagnosis of pneumonia made by the local treating physician during 14 days. Urinary tract infection and other infections were extracted from the serious adverse events registry.<br>Westendorp et al: assessed by physician and assessed by independent panel with infectious diseases specialists with modified CDC criteria during hospital admission.<br>Chamorro et al: infection was defined if temperature >37.5°C in 2 determinations or >37.8°C in a single determination in patients with suggestive symptoms (ie, cough, dyspnea, pleuritic pain, urinary tract symptoms), white blood cell count >11 000/mL or <4000/mL, pulmonary infiltrate on chest x-rays, or cultures positive for a pathogen. Otherwise, temperature >37.8°C was classified as noninfectious hyperthermia. Infection was further classified as early, if it occurred within the first 7 days after stroke, and late, when it supervened between days 8 and 90 after stroke. For the current analysis infection within 7 days was used.<br>Harms et al: modified CDC criteria within 11 days.<br>Chang et al: imaging, leukocytosis, fevers, clinical deterioration during follow-up (until 90 days)<br>Blacker et al: routine clinical diagnosis, rather than strict criteria; eg urine cultures showing white cells and bacteria in associated with appropriate clinical symptoms, or cough and sputum in association with changes seem on chest X-ray to diagnose respiratory infections, or skin redness, warmth and tenderness to diagnose cellulitis or phlebitis within 7 days post stroke.<br>Kohler et al: routine clinical diagnosis, rather than strict criteria; eg urine cultures showing white cells and bacteria in associated with appropriate clinical symptoms, or cough and sputum in association with changes seem on chest X-ray to diagnose respiratory infections, or skin redness, warmth and tenderness to diagnose cellulitis or phlebitis within 7 days post stroke.
Time to infection	Time to diagnosis of infection in days
Unfavorable outcome	Harms et al: mRS was not collected. Definition used: death or BI < 60.<br>For other trials: mRS 3-6.
Modified Rankin Scale Score	Kalra et al: assessed by by trial office researchers masked to allocation.<br>Westendorp et al: assessed by structured telephone interview by blinded trained trial team member.<br>Chamorro et al: -<br>Chang et al: assessed by neurologist<br>Fouda et al: primary clinical outcome was mRS at 90 days performed by co-investigator trained for mRS scoring.<br>Blacker et al: telephone interview by researchers from another hospital, blinded to the treatment allocation; standardized questionnaire to assess for recurrent vascular events; modified Rankin score to assess outcome; personnel were trained in modified Ranking

	scoring.<br>Kohler et al: telephone interview by researchers from another hospital, blinded to the treatment allocation; standardized questionnaire to assess for recurrent vascular events; modified Rankin score to assess outcome; personnel were trained in modified Ranking scoring.
Preventive antibiotic therapy name	When preventive antibiotic was given to a patient, name of antibiotic
Antibiotic therapy class	When preventive antibiotic was given to a patient, class of antibiotic. Class of antibiotic: 1=tetracyclin (minocyclin), 2=cefalosporins (ceftriaxone), 3=fluorchinolon (levofloxacin, moxifloxacin) 4= penicillin + macrolide (amoxicillin + clarithromycin)
Type of antibiotics	Kalra et al: treating physician was able to choose the type of antibiotic, but a recommendation was made for amoxicillin or co-amoxiclav, together with clarithromycin, these were named ‘trial antibiotics’. Patients were classified as having had penicillin + macrolide when these trial antibiotics were given at least 2 consecutive days in the first 4 days (regardless of other additional antibiotics). When no trial antibiotics were given, but other preventive antibiotics were given at least 2 times within the first 4 days, this was coded as ‘other antibiotics, type unknown’, since no data was present on what type of antibiotic was given when these were not the recommended trial antibiotics.<br>Other trials: one type of antibiotic was used for all patients randomized to preventive treatment, patients were classified in the corresponding group for the antibiotic (tetracyclins, cefalosporins, fluorchinolones, penicillin + macrolide).
Antibiotic therapy dose	When preventive antibiotic therapy was given to a patient, dose of antibiotic therapy per day in milligrams
Antibiotic therapy DDD	When preventive antibiotic therapy was given to a patient, the dose of antibiotic therapy converted to defined daily dosis (https://www.whocc.no/atc_ddd_index/)
Antibiotic therapy no of days	The number of days that each individual patient received *preventive* antibiotic therapy
Was the total treatment with preventive antibiotic therapy administered according to study protocol / per protocol	Whether each patient separately was treated according to the study protocol of the study in which patient was included:<br>- Kalra et al: in this trial antibiotic choice at intervention centres conformed to local antibiotic policy, but amoxicillin or co-amoxiclav, together with clarithromycin for 7 days were recommended if no restrictions applied. Treatment was considered as per protocol when the start of antibiotic therapy was initiated within 48 hours, and a patient was treated for at least 6 days of medication (day 0,2 and 4 or 2,4 and 6). If antibiotic therapy was stopped due to discharge or death before completing 6 days this was also considered treatment as per protocol.<br>- Westendorp et al: patients who received the complete 4 days treatment or patients who did not receive the complete 4 days treatment due to death, discharge or switch of antibiotic therapy due to infection were considered as being treated per protocol.<br>- Harms et al: patients were included in the per protocol analysis of the trial when they received the total treatment (eg patients who died within 11 days, were deblinded for medical reasons, were given less than 5 days study medication regardless of reason) were excluded. For the current analysis, per protocol was defined as total treatment but discontinuation of treatment due to death or infection was seen as treatment per protocol.<br>- Chamorro et al: days 500 mg/100 mL levofloxacin or an identical volume of placebo (0.9% physiological serum) intravenously, treatment was withdrawn in case of diagnosis of infection or death. For the current analysis, the patients that were excluded from per protocol population in the primary article were included in de per protocol population of this meta-analysis because treatment was discontinued due to prespecified reasons, eg infection, death or fever and this was considered as per protocol in the current meta-analysis.<br>- Chang et al: whether patients received the complete treatment. All patients did receive the complete treatment.<br>- Fouda et al: all patients randomized to minocyclin received the complete trial treatment the per protocol population is the same as the intention to treat population.<br>- Amiri-Nikpour et al: whether patients received the complete treatment, all patients did.<br>- Kohler et al: patients who received the complete treatment or patients who did not receive the complete treatment due to death or discharge.<br>- Blacker et al: patients who received the complete treatment or patients who did not receive

	the complete treatment due to death or discharge.
Time to first dose (hours)	Time in hours from stroke onset to administration of first dose of preventive antibiotic therapy.<br>- Kalra et al: data unavailable. Data available is whether a patient received medication within 24h or between 24 and 48h<br>- Westendorp et al: time in hours<br>- Harms et al: data unavailable<br>- Chamorro et al: data unavailable (was collected in trial but not able to retrieve data)<br>- Chang et al: in minutes, recalculated into hours<br>- Fouda et al: in minutes, recalculated into hours<br>- Blacker et al: in minutes, recalculated into hours<br>- Kohler: in minutes, recalculated into hours
A2ds2Totaal	Available for the following trials that included patients with ischemic stroke and in which the necessary variables were collected (age, atrial fibrillation, dysphagia, male sex, stroke severity): Kalra et al, Westendorp et al.
ISANtot	Available for the trials that collected the variables age, stroke severity and baseline modified Rankin Scale: Kalra et al, Westendorp et al, Chang et al, Blacker et al.
PASSpneutot	Available for the trials that collected age, sex, stroke severity, medical history of COPD and diabetes, baseline modified rankin scale, dysphagia: Kalra et al, Westendorp et al.
PASSinftot	Available for the trials that collected age, sex, stroke severity, medical history of diabetes, urinary catheter, baseline modified rankin scale: Westendorp et al, Chang et al.

Table S6. Baseline characteristics of type 1 trials		
**Characteristic**	**Preventive antibiotic treatment (n=1989)**	**Standard care / placebo (n=1981)**
**Age (years)**	75 (65-82)	76 (65-83)
**Male sex (%, n/N)**	52 (1032/1986)	52 (1031/1980)
**Medical history**		
COPD	9 (174/1988)	7 (143/1977)
Diabetes mellitus	19 (383/1988)	19 (379/1981)
Atrial fibrillation	22 (424/1919)	23 (446/1911)
**Pre-stroke disability (mRS)**	0 (0-1)	0 (0-1)
**Stroke severity (NIHSS)**	7 (4-15)	7 (4-15)
**Stroke type**		
- ischemic	85 (1693/1989)	86 (1711/1980)
- hemorrhagic	12 (229/1989)	10 (191/1980)
- TIA	2 (44/1989)	3 (50/1980)
- other diagnosis	1 (23/1989)	1 (28/1980)
Intravenous thrombolysis	32 (628/1950)	31 (605/1938)
**Dysphagia**	51 (922/1793)	51 (918/1795)
**Use of urinary cathether**	19 (252/1304)	21 (276/1305)
Data in % (n/N), median with interquartile range or mean with standard deviation<br>COPD = chronic obstructive pulmonary disease; mRS = modified Rankin Scale; NIHSS = National Institute of Stroke Severity Scale; TIA = Transient Ischemic Attack		

Table S7. Baseline characteristics of patients included in type 2 (Minocyclin) trials		
**Characteristic**	**Minocyclin (n=111)**	**Standard care / placebo (n=116)**
**Age (years)**	66 (58-75)	68 (57-75)
**Male sex (%, n/N)**	57 (63/111)	52 (60/116)
**Medical history**		
Obstructive pulmonary disease	3 (2/110)	9 (6/114)
Diabetes mellitus	32 (35/110)	36 (41/114)
Atrial fibrillation	16 (12/76)	21 (17/80)
**Pre-stroke disability (mRS)**	0 (0-0)	0 (0-0)
**Stroke severity (NIHSS)**	9 (6-13)	8 (5-13)
**Stroke type**		
- ischemic	79 (88/111)	76 (88/116)
- hemorrhagic	21 (23/111)	21 (24/116)
- TIA	0 (0/111)	3 (4/116)

- other diagnosis	0	0
Intravenous thrombolysis	28 (31/111)	25 (29/116)
**Dysphagia**	28 (31/111)	25 (29/116)
**Use of urinary cathether**		

Data in % (n/N), median with interquartile range or mean with standard deviation

COPD = chronic obstructive pulmonary disease; mRS = modified Rankin Scale; NIHSS = National Institute of Stroke Severity Scale; TIA = Transient Ischemic Attack

Table S8. Risk of bias assessment							
**First Author**	**RSG (Selection)**	**Allocation Concealment (Selection)**	**Blinding of participants (Performance)**	**Blinding of outcome (Detection)**	**Attrition Bias- incomplete outcome data**	**Reporting Bias**	**Other**
**Kalra et al, 2015**	Low	Low	(Open label) -High Risk for some selected outcomes	Low	Low	Low	Low
**Westendorp et al, 2015**	Low	Low	(Open label)- High risk for some selected outcome- ex. mRS/Infection	Low	Low	Low	Low
**Harms et al, 2008**	Low	Low	Low	Low	Unclear	Low	Low
**Chamorro et al, 2005**	Low	Low	Low	Low	Low	Low	Low
**Chang et al, 2017**	Low	Low	Low	Low	Low	Low	Low
**Fouda et al, 2017**	Unclear	Unclear	High	High	Low	Low	Low
**Amiri-Nikpour et al, 2015**	Unclear	Unclear	High	Low	High	Low	Low
**Kohler et al, 2015**	Low	Unclear	(Open label)- High risk for some selected outcomes	Low	Low	Low	Low
**Blacker et al, 2013**	Unclear	Unclear	Unclear	Unclear	Unclear	Unclear	Un-clear

**Table S9. Outcomes for each trial**								
	**3 Month mRS**	**3-month Survival**	**Discharge Death**	**Infe-Phys**	**Pneu-Phy**	**UTI-Phy**	**LOS**	**SAE**
**Kalra et al, 2015**	Yes	Yes	?	Yes	Yes	Yes	Yes	Yes
**Westendorp et al, 2015**	Yes	Yes	Yes	Yes	Yes	Yes	Yes	Yes
**Harms et al, 2008**	No-Only BI	Yes-6month	No	Yes (<11d)	Yes	No	No	Yes
**Chamorro et al, 2005**	Yes	Yes	No	Yes	Yes	Yes	No	No
**Chang et al, 2017**	Yes	No	No	No	No	No	Yes	Yes
**Fouda et al, 2017**	Yes	No	No	No	No	No	No	No
**Amiri-Nikpour et al, 2015**	No	Yes	No	No	No	No	No	No
**Kohler et al, 2015**	Yes	Yes (mRS 6)	No	No	No	No	No	No
**Blacker et al, 2013**	Yes	Yes	No	No	No	No	No	No

Table S10. Unfavorable outcome (mRS 3-6) at 3 months in all patients per trial					
**Trial**	**Preventive antibiotic therapy % n/N**	**Standard care % n/N**	**OR**	**95% CI**	**p-value**
Kalra et al	81.7 (486/595)	79.4 (465/586)	1.180	0.83-1.67	0.350
Chamorro et al	67.2 (45/67)	62.3 (43/69)	1.041	0.45-2.44	0.926
Westendorp et al	38.5 (484/1257)	39.9 (502/1257)	0.934	0.77-1.13	0.480
Harms et al	60 (18/30)	73.3 (22/30)	0.516	0.167-1.60	0.250
Chang et al	10 (1/10)	10 (1/10)	1.040	0.05-21.92	0.980
Kohler et al	34.1 (15/44)	29.8 (14/47)	2.163	0.49-9.55	0.308
Fouda et al*	66.7 (4/6)	85.7 (6/7)	0.481	0.12-1.95	0.305
Blacker et al	17.4 (4/23)	30.4 (7/23)	0.343	0.07-1.69	0.189
**Total**	**52.0 (1057/2032)**	**52.2 (1060/2029)**	**0.849**	**0.60-1.19**	**0.348**

CI denotes confidence interval, ref denotes reference category, NA not applicable.
Analysis adjusted for age and stroke severity. * for this trial only unadjusted analysis possible (number per events rule), it was excluded for the adjusted pooled analysis.

Figure S1. Forest plot for treatment effect per included trial on total range of mRS

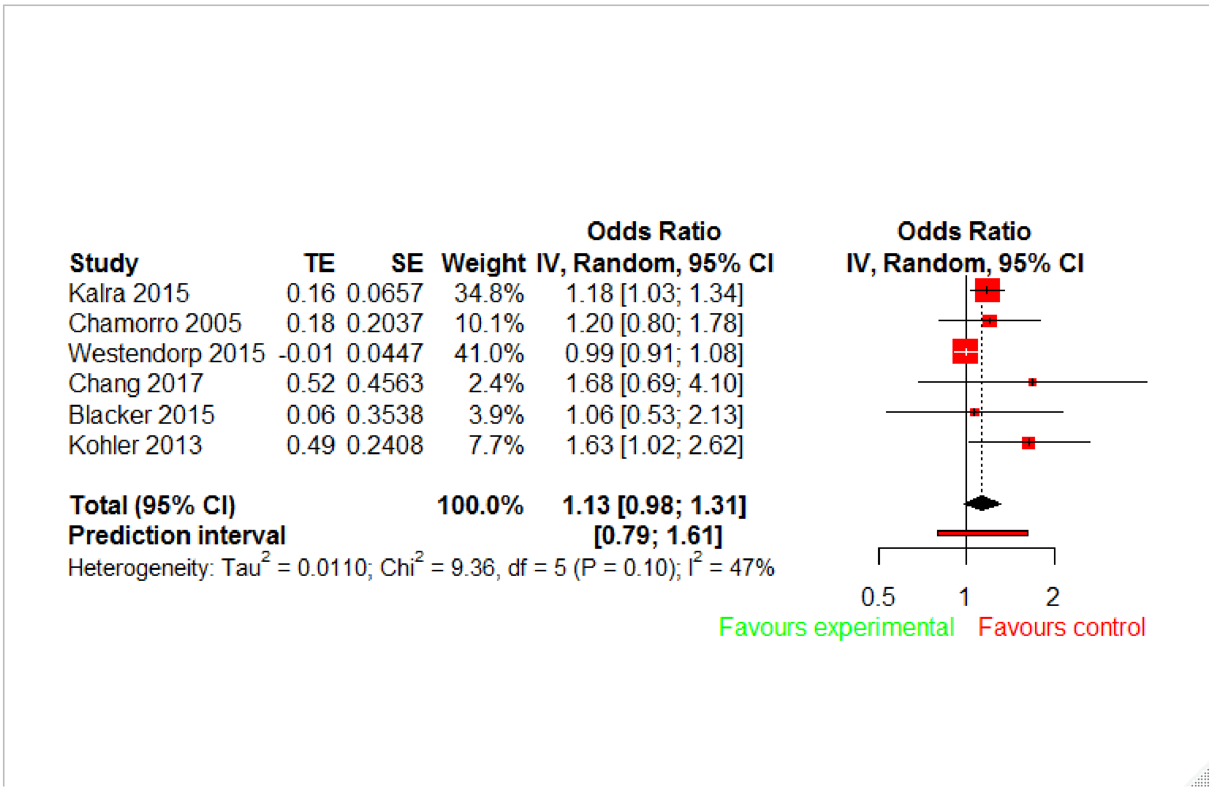

Table S11. Ordinal analysis mRS at 3 months in all patients per trial				
**Trial**	**No. of patients**	**OR***	**95% CI**	**p-value**
Kalra et al	1217	1.18	1.04-1.34	0.0129
Chamorro et al	136	1.20	0.80-1.78	0.3817
Westendorp et al	2538	0.99	0.91-1.08	0.8417
Harms et al	-	-	-	-
**Pool type 1**	**3891**	**1.08**	**0.94-1.25**	**0.2703**
Chang et al	20	1.68	0.69-4.10	0.2568
Kohler et al	91	1.63	1.02-2.62	0.0421
Fouda et al**	13	0.91	0.23-3.58	0.8916
Blacker et al	46	1.06	0.53-2.13	0.8639
**Pool type 2 adjusted**	157	1.46	1.02-2.09	0.0372
**Pool type 2 unadjusted**	**170**	1.23	0.88-1.74	0.2281
**All patients adjusted**	4061	1.13	0.98-1.31	0.0896
**All patients unadjusted**	4048	1.07	0.96-1.19	0.2226

CI denotes confidence interval, ref denotes reference category. * OR >1 favors control.

Adjusted for age and stroke severity (in categories)

** only unadjusted possible.

Supplementary figure 2. mRS scores at 3 months for all patients and for patients included in type 2 trials

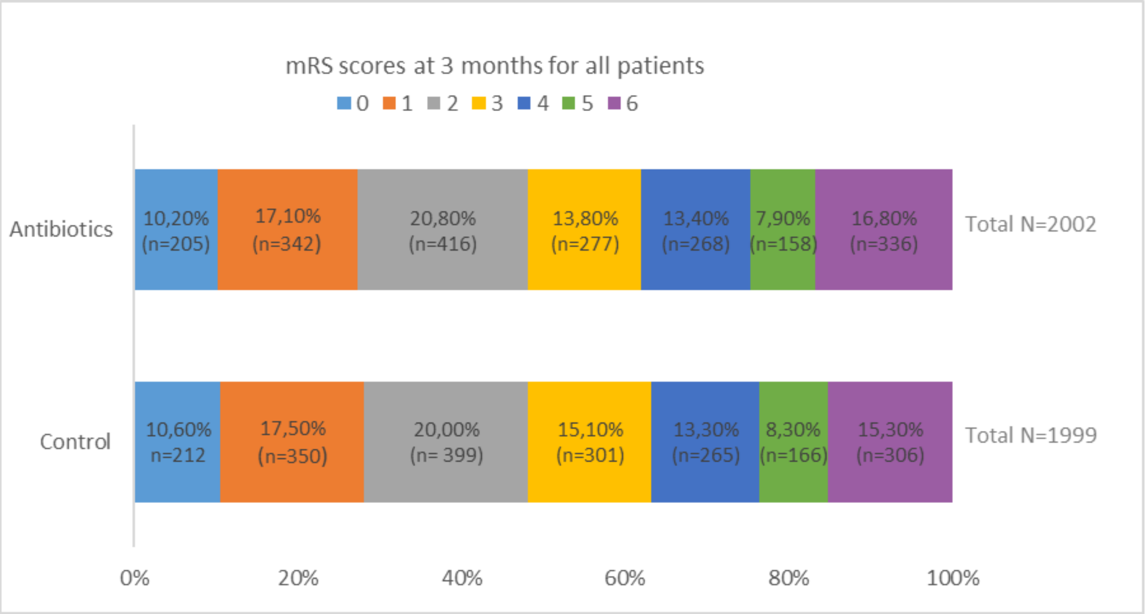

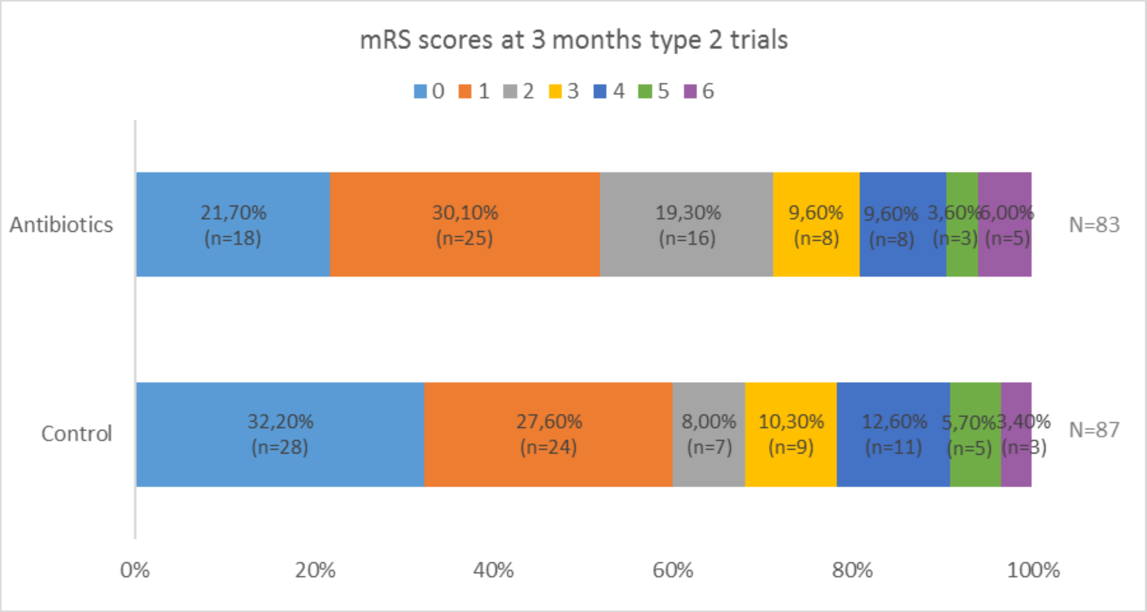

**Table S12.**<br>**Subgroup analyses for the primary outcome of functional outcome on total range of mRS for all trials**<br>The odds ratio represents the pooled odds ratio of the effect of antibiotic therapy vs standard therapy for each subgroup of patients within all trials. An odds ratio larger than one favors control/placebo					
**Subgroup**	**OR***	**95% CI**	**p-value**	**No pts**	**Trials included in analysis**
All patients adjusted	1.1318	0.9811-1.3056	0.0896	3968	Kalra, Westendorp, Chamorro, Chang, Blacker, Kohler.
All patients unadjusted	1.0684	0.9606-1.1883	0.2226	4001	All
Age >= 65	1.1061	0.9659-1.2667	0.1448	3012	Kalra, Westendorp, Chamorro, Blacker, Kohler.
Age >= 65 unadjusted	1.051	0.969-1.139	0.2278	3022	All
Age < 65	1.0217	0.8874-1.1763	0.7649	935	Kalra, Westendorp, Chamorro, Chang, Kohler.
Age < 65 unadjusted	1.006	0.875,1.157	0.9328	960	All
Age >= 75	1.1365	0.9628-1.3416	0.1305	2099	Kalra, Westendorp, Chamorro, Blacker, Kohler.
Age < 75	1.0425	0.9418-1.1540	0.4223	1867	Kalra, Westendorp, Chamorro, Chang, Blacker, Kohler.
Age < 75 unadjusted	1.082	0.977-1.198	0.1288	1878	All
Age >= 80	1.2099	0.9396-1.5579	0.1397	1414	Kalra, Westendorp, Chamorro, Blacker, Kohler.
Age >= 80 unadjusted	1.057	0.939-1.189	0.3582	1414	Kalra, Westendorp, Chamorro, Blacker, Kohler.
Age < 80	1.0206	0.9356-1.1133	0.6463	2560	Kalra, Westendorp, Chamorro, Chang, Blacker, Kohler.
Age < 80 unadjusted	1.035	0.948-1.129	0.4419	2570	All
NIHSS 0-5<br>NIHSS 6-42	0.9374<br>1.1091	0.8401-1.0459<br>1.0071-1.2215	0.2471<br>0.0354	1611<br>2375	Kalra, Westendorp, Chang, Kohler<br>Kalra, Westendorp, Chamorro, Chang, Blacker, Kohler.
NIHSS 0-5 unadjusted	0.931	0.834-1.039	0.2001	1600	Kalra, Westendorp, Chamorro, Chang, Blacker, Kohler.
NIHSS 6-42	1.095	0.999-1.199	0.0513	2387	All
NIHSS 0-10<br>NIHSS 11-42	1.0937<br>1.1513	0.9152-1.3071<br>1.0278- 1.2896	0.3245<br>0.0149	2507<br>1480	Kalra, Westendorp, Chamorro, Chang, Kohler.<br>Kalra, Westendorp, Chamorro, Chang, Blacker, Kohler.
NIHSS 0-10 unadjusted	1.013	0.927-1.106	0.7814	2512	All
NIHSS 11-42 unadjusted	1.095	0.977-1.227	0.1183	1488	All
Iv-thrombolysis yes<br>Iv-thrombolysis no<br>Iv-thrombolysis no unadjusted	0.9510<br>-<br>1.1070	0.7768-1.1643<br>-<br>0.9858-1.2432	0.6266<br>-<br>0.0858	1262<br>-<br>2155	Kalra, Westendorp, Blacker<br>Adjusted only data from type 1 trials<br>Kalra, Westendorp, Kohler
Ischemic stroke	1.0931	0.9551- 1.2509	0.1959	3385	Kalra, Westendorp, Chamorro, Blacker, Kohler

Hemmorrhagic stroke	1.0731	0.7835-1.4699	0.6602	420	Kalra, Westendorp, Chamorro, Kohler
Ischemic stroke unadjusted	1.026	0.951-1.107	0.5123	3386	All except Chang, Fouda
Hemorrhagic stroke unadjusted	1.052	0.853-1.299	0.6346	444	All except Blacker
Time to therapy <24h<br>Time to therapy > 24h	1.1202<br>-	0.9464-1.3258<br>-	0.1870<br>-	2250<br>-	Kalra, Westendorp, Chang, Blacker, Kohler<br>Only data from type 1 trial
Time to therapy < 24h unadjusted	0.9950	0.9245-1.0708	0.8934	2267	Kalra, Westendorp, Fouda, Chang, Blacker, Kohler
Time to therapy:<br>- 0-5<br>- 6-11<br>- 12-24	 0.9225<br>1.6138<br>-	 0.7854-1.0836<br>0.5419-4.8063<br>-	 0.3258<br>0.3901<br>-	 1525<br>1746<br>-	 Westendorp, Blacker<br>Westendorp, Kohler<br>Only data from type 1 trial
Treatment per protocol	1.1475	0.8271-1.5920	0.4100	2685	Westendorp, Chang, Blacker, Kohler
Placebo controlled<br>Open label unadjusted<br>Open label adjusted	1.2643<br>1.0435<br>1.1196	0.8781-1.8204<br>0.9387- 1.1599<br>0.9472-1.3233	0.2073<br>0.4306<br>0.1856	156<br>3905<br>3892	Chamorro, Chang<br>Westendorp, Kalra, fouda, Blacker, Kohler<br>Westendorp, Kalra, Blacker, Kohler

Table S13.

Subgroup analyses for the primary outcome of functional outcome on total range of mRS for type 1 trials

The odds ratio represents the pooled odds ratio of the effect of antibiotic therapy vs standard therapy for each subgroup of patients within type 1 trials. An odds ratio larger than one favors control/placebo

**Subgroup**	**OR**	**95% CI**	**p-value**	**No. of pts**	
All patients unadjusted	1.0731	0.9178-1.2546	0.3767	3831	Kalra, Westendorp, Chamorro
All patients adjusted	1.0834	0.9396-1.2493	0.2703	3812	Kalra, Westendorp, Chamorro
Age >= 65	1.0913	0.9337-1.2755	0.2724	2927	Kalra, Westendorp, Chamorro
Age < 65	1.0200	0.8412-1.2368	0.8404	885	Kalra, Westendorp, Chamorro
Age >= 75	1.1091	0.9527 - 1.2911	0.1819	2049	Kalra, Westendorp, Chamorro
Age < 75	1.0437	0.9086-1.1990	0.5454	1763	Kalra, Westendorp, Chamorro
Age >= 80	1.1096	0.9864-1.2482	0.0832	1383	Kalra, Westendorp, Chamorro
Age < 80	1.0342	0.8985-1.1904	0.6393	2434	Kalra, Westendorp, Chamorro
NIHSS 0-5<br>NIHSS 6-42	0.9456<br>1.1214	0.7826-1.1425<br>0.9684-1.2986	0.5619<br>0.1258	1554<br>2275	Kalra, Westendorp, Chamorro<br>Kalra, Westendorp, Chamorro
NIHSS 0-10<br>NIHSS 11-42	0.9986<br>1.1279	0.9127-1.0925<br>0.9860-1.2902	0.9754<br>0.0794	2401<br>1429	Kalra, Westendorp, Chamorro<br>Kalra, Westendorp, Chamorro
Iv-thrombolysis yes<br>Iv-thrombolysis no	0.9478<br>1.1085	0.7370-1.2189<br>1.0092-1.2176	0.6762<br>0.0314	1216<br>2092	Kalra, Westendorp<br>Kalra, Westendorp, Chamorro
Ischemic stroke	1.0690	0.9270-1.2328	0.3590	3263	Kalra, Westendorp, Chamorro

Hemmorrhagic stroke<br>TIA	1.0153<br>0.977	0.7193-1.4330<br>0.598-1.595	0.9312<br>0.9257	400<br>86	Kalra, Westendorp, Chamorro<br>Westendorp
Dysphagia yes<br>Dysphagia no	1.1239<br>0.971	1.0002-1.2628<br>0.874-1.078	0.0496<br>0.5791	1780<br>1731	Kalra, Westendorp<br>Westendorp
A2ds2-score > 5	1.0517	0.8676-1.2748	0.6077	1360	Kalra, Westendorp
A2ds2-score <= 5	0.9905	0.8896-1.1028	0.8613	1667	Kalra, Westendorp
PASS inf score>10<br>PASS inf score <=10	1.165<br>0.971	0.931-1.458<br>0.880-1.072	0.1810<br>0.5640	376<br>1949	Westendorp
ISAN-score >10<br>ISAN-score < 10	1.031<br>0.980	0.850-1.250<br>0.889-1.081	0.7550<br>0.6880	516<br>1995	Westendorp<br>Westendorp
Time to therapy < 24h<br>Time to therapy >= 24h	1.0579<br>1.3908	0.8884-1.2598<br>0.7798-2.4807	0.5275<br>0.2638	2097<br>1958	Kalra, Westendorp<br>Kalra, Westendorp
Time to therapy:<br>- < 6<br>- 6-12<br>- 12-24	 0.916<br>0.957<br>1.004	 0.776-1.080<br>0.847-1.083<br>0.897-1.125	 0.2955<br>0.4866<br>0.9382	 1482<br>1682<br>1771	 Westendorp<br>Westendorp<br>Westendorp
Type of antibiotic:<br>- tetracyclin: see analysis type 2 trials<br>- cephalosporin<br>- fluorchinolones<br>- penicillin + macrolide	 <br>0.991<br>1.195<br>1.132	 <br>0.908-1.082<br>0.802-1.782<br>0.991-1.293	 <br>0.8417<br>0.3817<br>0.0673	 <br>2538<br>135<br>1093	 <br>Westendorp<br>Chamorro<br>Kalra
Treatment per protocol	0.954	0.872-1.045	0.3110	2353	Westendorp
Placebo controlled study<br>Open label study	1.195<br>1.0728	0.802-1.782<br>0.9068-1.2691	0.3817<br>0.4127	136<br>3755	Chamorro<br>Westendorp, Kalra
* OR > 1 favors control/placebo					

Table S14.

Subgroup analyses for the primary outcome of functional outcome on total range of mRS for type 2 trials

The odds ratio represents the pooled odds ratio of the effect of antibiotic therapy vs standard therapy for each subgroup of patients within type 2 trials. An odds ratio larger than one favors control/placebo

Subgroup	OR	95% CI	p-value	No. pts	Included trials in analysis
All patients adjusted	1.4626	1.0229-2.0914	0.0372	156	Chang, Blacker and Kohler
All patients unadjusted	1.2348	0.8762-1.7401	0.2281	170	Chang, Fouda, Blacker, Kohler
Age >= 65	1.4133	0.8677-2.3018	0.1646	85	Blacker and Kohler
Age >= 65 unadjusted	1.3551	0.8543-2.1494	0.1967	95	Chang, Fouda, Blacker, Kohler
Age < 65	1.4098	0.7827-2.5394	0.2527	50	Chang and Kohler
Age < 65 unadjusted	1.2765	0.7658-2.1277	0.3491	75	Chang, Fouda, Blacker, Kohler
Age >= 75	1.5755	0.6194-4.0073	0.3399	50	Blacker and Kohler
Age >= 75 unadjusted	1.1468	0.6043-2.1762	0.6752	50	Blacker, Kohler
Age < 75	1.2919	0.8481-1.9678	0.2330	104	Chang, Blacker and Kohler
Age < 75 unadjusted	1.3362	0.8791-2.0308	0.1748	115	Chang, Fouda, Blacker, Kohler
Age >= 80	1.8129	0.2501-	0.5561	31	Blacker, Kohler

		13.1400			
Age >= 80 unadjusted	1.2352	0.5438-2.8058	0.6138	31	Blacker, Kohler
Age < 80	1.2361	0.8382-1.8230	0.2848	126	Chang, Blacker, Kohler
Age < 80 unadjusted	1.2461	0.8454-1.8368	0.2664	136	Chang, Fouda, Blacker, Kohler
NIHSS 0-5	1.4719	0.8007-2.7057	0.2133	57	Chang, Kohler
NIHSS 0-5 unadjusted	1.3872	0.7900-2.4359	0.2545	46	Chang, Blacker, Kohler
NIHSS 6-42	1.1046	0.6938-1.7588	0.6749	100	Chang, Blacker, Kohler
NIHSS 6-42 unadjusted	1.0797	0.7023-1.6597	0.7268	112	Chang, Fouda, Blacker, Kohler
NIHSS 0-10	1.3453	0.8207-2.2054	0.2395	106	Chang, Blacker, Kohler
NIHSS 0-10 unadjusted	1.2382	0.7666-2.0000	0.3825	111	Chang, Fouda, Blacker, Kohler
NIHSS 11-42	1.8148	0.9370-3.5149	0.0772	51	Chang, Blacker, Kohler
NIHSS 11-42 unadjusted	1.3673	0.7566-2.4708	0.3002	59	Chang, Fouda, Blacker, Kohler
Iv-thrombolysis yes	1.063	0.531-2.125	0.8639	46	Blacker
Iv-thrombolysis yes unadjusted	0.9781	0.5438-1.7594	0.9412	60	Blacker, Kohler
Iv-thrombolysis no	1.460	0.843-2.529	0.1763	63	Kohler
IV-thrombolysis no unadjusted	1.422	0.814-2.487	0.2162	63	Kohler
Ischemic stroke	1.3518	0.8949-2.0420	0.1521	122	Blacker, Kohler
Ischemic stroke unadjusted	1.2064	0.8065-1.8045	0.3610	123	Blacker, Kohler
Hemmorrhagic stroke	1.678	0.686-4.104	0.2568	20	Chang
Hemmorrhagic stroke unadjusted	1.2506	0.6260-2.4984	0.5265	44	Chang, Fouda, Kohler
Time to therapy < 24h	1.4465	1.0059-2.0800	0.0464	153	Chang, Blacker, Kohler
Time to therapy < 24h unadjusted	1.2001	0.8497-1.6950	0.3005	170	Chang, Fouda, Blacker, Kohler
Time to therapy >= 24h	NA	NA	NA	NA	NA
Time to therapy 0-6h<br>Time to therapy 0-6h unadjusted	1.049<br>0.9348	0.518-2.121<br>0.6194-1.4108	0.8951<br>0.7482	43<br>122	Blacker<br>Chang, Fouda, Blacker, Kohler
Time to therapy 6-12h	2.921	1.661-5.136	0.0002	64	Kohler
Time to therapy 6-12h unadjusted	1.1637	0.4896-2.7659	0.7314	117	Chang, Fouda, Blacker, Kohler
Time to therapy 12-24h	NA	NA	NA	NA	-
Time to therapy 12-24h unadjusted	1.2547	0.5553-2.8351	0.5853	81	Chang, Fouda, Kohler
Treatment per protocol	1.4105	0.9836-2.0227	0.0615	150	Chang, Blacker, Kohler
Treatment per protocol unadjusted	1.1353	0.8021-1.6069	0.4739	163	Chang, Fouda, Blacker, Kohler
Treatment not per protocol	NA	NA	NA	NA	(only 3 patients in trial Kohler, all randomization=1)
Treatment not per protocol unadjusted	NA	NA	NA	NA	(only 3 patients in trial Kohler, all randomization=1)
Placebo controlled study<br>Open label study unadjusted<br>Open label study adjusted	1.678<br>1.1975<br>1.4245	0.686-4.104<br>0.8300- 1.7278<br>0.9634-2.1061	0.2568<br>0.3352<br>0.0762	20<br>137<br>150	Chang<br>Blacker, Kohler<br>Fouda, Blacker, Kohler

Table S15.

Subgroup analyses for the primary outcome of unfavorable functional outcome (mRS 3-6) for all trials.

The odds ratio represents the interaction effect (the effect of antibiotic treatment vs placebo/standard care compared between both subgroups) the p-value represents the p-value for interaction.

Subgroup	Preventive antibiotic therapy % n/N	Standard care % n/N	OR	95% CI	p-value interaction
All patients unadjusted	52.0 (1057/2032)	52.2 (1060/2029)	1.160	0.87-1.55	0.312
All patients adjusted	52.0 (1057/2032)	52.2 (1060/2029)	1.178	0.84-1.66	0.348
Age per year	NA	NA	1.003	0.99-1.02	0.681
Age<br>- 0 - 50<br>- 51 – 60<br>- 61 – 70<br>- 71 – 80<br>- 81 – 90<br>> 91	 32.5 (37/114)<br>32.3 (74/229)<br>38.9 (159/409)<br>50.0 (313/626)<br>69.7 (381/547)<br>86.9 (93/107)	 30.3 (37/122)<br>31.4 (74/236)<br>37.2 (136/366)<br>53.5 (345/645)<br>68.7 (388/565)<br>84.2 (80/95)	 1.019	 0.89-1.17	 0.779
Age <= 65<br>Age > 65	32.9 (177/538)<br>58.9 (880/1494)	33.0 (174/528)<br>59.0 (886/1501)	0.946	0.66-1.36	0.765
Age <= 75<br>Age > 75	39.5 (409/1035)<br>65.0 (648/997)	37.4 (378/1011)<br>67.0 (682/1018)	1.082	0.9-1.49	0.624
Age <= 80<br>Age > 80	42.3 (583/1378)<br>72.5 (474/654)	43.2 (592/1369)<br>70.9 (468/660)	0.842	0.60-1.19	0.328
Stroke severity per point on NIHSS	NA	NA	1.007	0.97-1.04	0.684
Stroke severity (NIHSS):<br>- 0 – 5<br>- 6 – 10<br>- 10 – 20<br>- 20 - 42	 20.8 (167/801)<br>49.0 (223/455)<br>82.8 (466/563)<br>94.3 (199/211)	 24.8 (202/813)<br>44.9 (199/443)<br>81.9 (465/568)<br>94.6 (192/203)	 1.201	 0.99-1.46	 0.063
NIHSS 0-5<br>NIHSS 6-42	20.8 (167/801)<br>72.3 (888/1229)	24.8 (202/813)<br>70.5 (856/1214)	0.703	0.51-0.97	0.033
NIHSS 0-10<br>NIHSS 11-42	31.1 (390/1256)<br>85.9 (665/774)	31.9 (401/1256)<br>85.2 (657/771)	0.798	0.55-1.16	0.242
Iv-thrombolysis yes<br>Iv-thrombolysis no	47.1 (301/639)<br>54.2 (570/1052)	53.9 (327/607)<br>51.0 (562/1101)	0.720	0.50-1.04	0.083
Dysphagia yes<br>Dysphagia no	78.0 (702/900)<br>25.1 (216/862)	76.6 (688/898)<br>27.3 (237/869)	1.294	0.82-2.05	0.274
Stroke type<br>- ischemic<br>- hemorrhagic<br>- TIA<br>- other diagnosis	 51.7 (889/1721)<br>64.2 (158/246)<br>11.9 (5/42)<br>21.7 (5/23)	 52.4 (913/1741)<br>62.1 (128/206)<br>20 (10/50)<br>25.8 (8/31)	 1.052<br>1.083<br>0.674<br>ref	 0.24-4.58<br>0.23- 5.07<br>0.10-4.70<br>ref	 0.947<br>0.919<br>0.691<br>0.924
Stroke type<br>- ischemic<br>- hemorrhagic	 51.7 (889/1721)<br>64.2 (158/246)	 52.4 (913/1741)<br>62.1 (128/206)	 0.971	 0.57-1.65	 0.914
Time to therapy <= 24h<br>Time to therapy > 24h	48.8 (844/1731)<br>80.6 (104/129)	NA	0.684	0.39-1.19	0.179
Time to therapy:					

- 0-6<br>- 7-12<br>- 13-18<br>- 19-24<br>- > 24	35.1 (129/368)<br>37.7 (170/451)<br>34.3 (79/230)<br>42.6 (92/216)<br>80.6 (104/129)	NA	1.147	1.03-1.28	0.016
Type of antibiotic:<br>- tetracyclin<br>- cephalosporin<br>- fluorchinolones<br>- penicillin + macrolide	 28.9 (24/83)<br>38.5 (484/1257)<br>67.2 (45/67)<br>80.9 (424/524)	 NA	 0.549<br>0.726<br>0.569<br>ref	 0.02-16.52<br>0.28-1.90<br>0.26-1.27<br>ref	 0.730<br>0.514<br>0.168<br>0.445
Treatment per protocol<br>Treatment not pp	49.7 (855/1722)<br>64.8 (193/298)	52.1 (1049/2012)<br>64.7 (11/17)	1.269	0.38-4.22	0.697
Placebo controlled<br>Open label	59.8 (64/107)<br>51.6 (993/1925)	60.6 (66/109)<br>51.8 (994/1920)	1.065<br>1.178	0.47-2.41<br>0.84-1.66	0.880<br>0.347
All analyses corrected for age and stroke severity.<br>CI denotes confidence interval, ref denotes reference category, NA not applicable.					

Table S16.

Subgroup analyses for the primary outcome of unfavorable functional outcome (mRS 3-6) for type 1 trials

The odds ratio represents the interaction effect (the effect of antibiotic treatment vs placebo/standard care compared between both subgroups) the p-value represents the p-value for interaction.

Subgroup	Preventive antibiotic therapy % n/N	Standard care % n/N	OR	95% CI	p-value interaction
All patients unadjusted	53 (1033/1949)	53.1 (1032/1942)	1.160	0.87-1.55	0.312
All patients adjusted	53 (1033/1949)	53.1 (1032/1942)	1.177	0.84-1.66	0.348
Age per year	NA	NA	1.002	0.99-1.02	0.728
Age<br>- 0 - 50<br>- 51 – 60<br>- 61 – 70<br>- 71 – 80<br>- 81 – 90<br>> 91	 33.7 (35/104)<br>33.3 (71/213)<br>39.7 (153/385)<br>50.5 (306/606)<br>70.2 (375/534)<br>86.9 (93/107)	 31.5 (35/111)<br>32.3 (70/217)<br>36.8 (127/345)<br>54 (339/628)<br>69.8 (381/546)<br>84.2 (80/95)	 1.107	 0.89-1.17	 0.812
Age <= 65<br>Age > 65	33.8 (168/497)<br>59.6 (865/1452)	33.2 (162/488)<br>59.8 (870/1454)	1.019	0.70-1.48	0.923
Age <= 75<br>Age > 75	40.5 (394/974)<br>65.5 (639/975)	38.0 (361/951)<br>67.7 (671/991)	1.097	0.80-1.51	0.570
Age <= 80<br>Age > 80	43.2 (565/1308)<br>73.0 (468/641)	43.9 (571/1301)<br>71.9 (461/641)	0.859	0.61-1.22	0.393
Stroke severity per point on NIHSS	NA	NA	1.009	0.98-1.04	0.619
Stroke severity (NIHSS):<br>- 0 – 5<br>- 6 – 10	 21.4 (166/775)<br>51.1 (218/427)	 25.6 (200/781)<br>46.2(193/418)	 1.202	 0.99-1.46	 0.065

- 10 – 20<br>- 20 - 42	83.9 (455/542)<br>94.4 (187/198)	82.9 (450/543)<br>94.6 (192/203)			
NIHSS 0-5<br>NIHSS 6-42	21.4 (166/775)<br>73.8 (865/1172)	25.6 (200/781)<br>71.6 (830/1159)	0.692	0.50-0.96	0.028
NIHSS 0-10<br>NIHSS 11-42	31.9 (384/1202)<br>86.8 (647/745)	32.8 (393/1199)<br>86.0 (637/741)	0.813	0.55-1.19	0.291
Iv-thrombolysis yes<br>Iv-thrombolysis no	48.2 (293/608)<br>55 (562/1021)	54.7 (316/578)<br>52 (556/1069)	0.727	0.50-1.06	0.094
Dysphagia yes<br>Dysphagia no	78 (702/900)<br>25.1 (216/862)	76.6 (688/898)<br>27.3 (237/869)	1.294	0.82-2.05	0.274
Stroke type<br>- ischemic<br>- hemorrhagic<br>- TIA<br>- other diagnosis	 52.6 (873/1659)<br>66.7 (150/225)<br>11.9 (5/42)<br>21.7 (5/23)	 53.3 (896/1680)<br>63.9 (117/183)<br>20.0 (10/50)<br>28.6 (8/28)	 ref<br>1.061<br>0.643<br>0.901	 ref<br>0.62-1.81<br>0.18-2.35<br>0.21-3.95	 0.913<br>0.829<br>0.504<br>0.890
Stroke type<br>- ischemic<br>- hemorrhagic	 52.6 (873/1659)<br>66.7 (150/225)	 53.3 (896/1680)<br>63.9 (117/183)	 0.943	 0.55-1.61	 0.831
A2ds2-score 0-3<br>A2ds2-score 4-6<br>A2ds2-score 7-10	20.0 (109/546)<br>50.3 (231/459)<br>87.2 (431/494)	22.7 (127/559)<br>51.3 (242/472)<br>86.7 (435/502)	1.067	0.81-1.40	0.644
A2ds2-score 0-5<br>A2ds2-score 5-10	27.1 (223/824)<br>81.2 (548/675)	28.2 (238/843)<br>82.0 (566/690)	1.096	0.71-1.70	0.682
ISAN-score 0-10<br>ISAN-score > 10	35.4 (436/1233)<br>86.1 (520/604)	35.8 (440/1230)<br>86.1 (520/604)	1.023	0.68-1.55	0.916
ISAN-score 0-7<br>ISAN-score 8-15<br>ISAN-score 16-21	21.3 (158/743)<br>68.5 (636/929)<br>98.2 (162/165)	23.4 (178/760)<br>68.5 (640/929)<br>97.9 (142/145)	1.093	0.78-1.52	0.601
Time to therapy < 24h<br>- <= 24h<br>- > 24h	 49.8 (824/1653)<br>80.5 (103/128)	 NA<br>NA	 0.756	 0.43-1.33	 0.331
Time to therapy:<br>- 0-6<br>- 7-12<br>- 13-18<br>- 19-24<br>- > 24	 36.6 (120/328)<br>38.6 (166/430)<br>34.4 (76/221)<br>42.3 (88/208)<br>80.5 (103/128)	 NA	 1.092	 0.96-1.24	 0.168
Treatment per protocol<br>- no<br>- yes	 65.0 (191/294)<br>50.7 (835/1646)		 1.247	 0.38-4.13	 0.718
Placebo controlled<br>Open label	64.9 (63/97)<br>52.4 (970/1825)	65.7 (65/99)<br>52.5 (967/1843)	1.178<br>1.061	0.84-1.66<br>0.47-2.42	0.347<br>0.887
All analyses corrected for age and stroke severity.<br>CI denotes confidence interval, ref denotes reference category, NA not applicable.					

Table S17.

Subgroup analyses for the primary outcome of unfavorable functional outcome (mRS 3-6) for type 2 trials

The odds ratio represents the interaction effect (the effect of antibiotic treatment vs placebo/standard care compared between both subgroups) the p-value represents the p-value for interaction.

Subgroup	Preventive antibiotic therapy % n/N	Standard care % n/N	OR	95% CI	p-value interaction
All patients adjusted	28.9 (24/83)	32.2 (28/87)	0.245	0.04-1.44	0.120
All patients unadjusted	28.9 (24/83)	32.2 (28/87)	0.481	0.12-1.95	0.305
Age per year	NA	NA	1.010	0.93-1.10	0.811
Age<br>- 0 - 50<br>- 51 – 60<br>- 61 – 70<br>- 71 – 80<br>- 81 – 90<br>> 91	 20 (2/10)<br>18.8 (3/16)<br>25 (6/24)<br>35.0 (7/20)<br>46.2 (6/13)<br>-	 18.2 (2/11)<br>21.1 (4/19)<br>42.9 (9/21)<br>35.3 (6/17)<br>36.8 (7/19)<br>-	 1.015	 0.48-2.17	 0.969
Age <= 65<br>Age > 65	22.0 (9/41)<br>35.7 (15/42)	30.0 (12/40)<br>34.0 (16/47)	0.332	0.05-2.47	0.282
Age <= 75<br>Age > 75	24.6 (15/61)<br>40.9 (9/22)	28.3 (17/60)<br>40.7 (11/27)	0.767	0.11-5.24	0.787
Age <= 80<br>Age > 80	25.7 (18/70)<br>46.2 (6/13)	30.9 (21/68)<br>36.8 (7/19)	0.497	0.06-4.27	0.524
Stroke severity per point on NIHSS	NA	NA	0.980	0.80-1.20	0.842
Stroke severity (NIHSS):<br>- 0 – 5<br>- 6 – 10<br>- 10 – 20<br>- 20 - 42	 3.8 (1/26)<br>17.9 (5/28)<br>52.4 (11/21)<br>85.7 (6/7)	 6.3 (2/32)<br>24 (6/25)<br>60.0 (15/25)<br>100 (5/5)	 1.431	 0.38-5.45	 0.600
NIHSS 0-5<br>NIHSS 6-42	3.8 (1/26)<br>40.4 (23/57)	6.3 (2/32)<br>47.3 (26/55)	0.891	0.06-13.68	0.934
NIHSS 0-10<br>NIHSS 11-42	11.1 (6/54)<br>62.1 (18/29)	14 (8/57)<br>66.7 (20/30)	0.386	0.05-3.10	0.370
Iv-thrombolysis yes<br>Iv-thrombolysis no	25.8 (8/31)<br>25.8 (8/31)	37.9 (11/29)<br>18.8 (6/32)	0.437	0.01-13.41	0.635
Ischemic stroke<br>Hemmorrhagic stroke	25.8 (16/62)<br>38.1 (8/21)	27.9 (17/61)<br>47.8 (11/23)	0.347	0.01-12.27	0.561
Time to therapy:<br>- 0-6<br>- 7-12<br>- 13-18<br>- 19-24	 22.5 (9/40)<br>19.0 (4/21)<br>33.3 (3/9)<br>50.0 (4/8)	 NA	 2.30	 1.11-4.76	 0.025

- > 24	100 (1/1)				
Treatment per protocol<br>- no<br>- yes	 50.0 (2/4)<br>26.3 (20/76)		7.075	0.15-326.51	0.317
Placebo controlled	10 (1/10)	10 (1/10)	1.040	0.05-21.92	0.980
Open label	31.5 (23/73)	35.1 (27/77)	1.739	0.48-6.29	0.399
All analyses corrected for age and stroke severity.<br>CI denotes confidence interval, ref denotes reference category, NA not applicable.					

Supplementary table 18. Adverse events									
	**Kalra et al, 2015**	**Westen-dorp et al, 2015**	**Harms et al, 2008**	**Chamorro et al, 2005**	**Chang et al, 2017**	**Fouda et al, 2017***	**Amiri-Nikpour et al, 2015****	**Kohler et al, 2015**	**Blacker et al, 2013**
Neurological									
**CT confirmed stroke extension**	23/615 (4%) vs 22/602 (4%)	-	-	-	0 vs 0	-	-	1 (2.1%) vs 0	-
**Recurrent stroke**	-	-	1 (2.6%) vs 0	-	0 vs 0	1 vs 0	0 vs 0	0 vs 1 (2.2%)	-
**Hemmorrhagic trans-formation**	-	-	2 (5.1%) vs 1 (2.5%)	-	-	-	-	1 (2.1%) vs 1 (2.1%)	-
**Other neurological events**	14/615 (2%) vs 12/602 (2%)	-	-	-	-	-	-	-	-
General									
**Gastrointestinal bleed**	5/615 (0.8%) vs 6/602 (1%)	-	-	-	-	-	-	-	-
**Gastrointestinal other**	-	-	3 (7.7%) vs 4 (10%)	-	-	-	-	1 (2.1%) vs 0	-
**Cardiac (MI, HF, pulmonary edema)**	15/615 (2%) vs. 11/602 (2%)	-	1 (2.6%) vs 1 (2.5%)	-	-	-	-	-	-
**Pulmonary adverse event**	-	-	0 vs 3 (7.5%)	-	-	-	-	-	-
**Transfer to ICU**	6/615 (1%) vs 4/602 (0.7%)	-	-	-	-	-	-	-	-
Development of antibiotic resistance									
**Infection and/or colonization with resistant organism**	11/615 (2%) vs. 14/602 (2%) (MRSA colonisation)	7 (0.6%) vs 5 (0.4%) (ceftriaxone resistant infection)	1 vs 1 (*E coli* from stool sample resistant to ciprofloxacin and moxifloxa-cin) 1 vs 0 MRSA isolates (but present before start therapy	-	0 vs 0	-	-	-	-
Side effects of medication									
**Allergic reaction to antibiotic**	-	6 (0.5) vs 5 (0.4)	-	-	0 vs 0	-	-	1 (2.1%) vs 1 (2.2%) (rash)	-
**Diarrhoea by C difficile**	2/615 (0.3%) vs 4/602 (0.7%)	2 (<0.2) vs 0	-	-	0 vs 0	-	-	-	-
**Phlebitis**	-	15 (1.2 vs 0.7) vs 9	-	-	-	-	-	-	-
**Raised liver enzymes**	Both: 8/615 (1%) vs	152 (12%) vs 129 (10%)	1 (2.6%) vs 0	-	-	-	-	4 (8.5%) vs 2 (4.2%)	-

**Oliguria or raised plasma enzymes**	7/602 (1%)	101 (8%) vs 112 (9%)	1 (2.6%) vs 0	-	-	-	-	Mean creatinine at day 7 similar in both groups	-
**Renal failure**	-	-	-	-	-	-	-	0 vs 1 (2.1%)	-
**Other**									
**Miscellaneous**	6/615 (1%) vs. 8/602 (1%)	-	3 (7.7%) vs 0	-	1 (5%) vs 0 (nausea)	-	-	3 (7.1%) vs 2 (4.4%) (headache)	-
Data reported as no. (%) of patients randomized to antibiotic therapy vs. no. (%) patients randomized to placebo/ standard care<br>* Fouda et al: 'minocycline was well tolerated', study did not mention adverse events separately.<br>** Amiri-Nikpour et al: 'During 90-day follow-up, no adverse outcomes including myocardial infarction, recurrent stroke, and mortality were observed in the both groups', study did not mention adverse events separately.									

Supplementary table 19. Analysis of unfavorable outcome with NIHSS 5 cutoff for type 1 trials separately

		**NIHSS <5**				**NIHSS >=5**		
		**AB**	**control**			**AB**	**control**	
**Study**								
**Kalra et al**	mRS 0-2	68% (48)	63% (42)	90		12% (61)	15% (79)	140
	mRS 3-6	32% (23)	37% (25)	48		88% (461)	85% (438)	899
		71	67	138		522	517	1039
OR 1.656, 95%CI 0.75-3.67; p-value interaction 0.213								
**Chamorro et al**	mRS 0-2	57% (4)	57% (4)	8		30% (18)	36% (22)	40
	mRS 3-6	43% (3)	43% (3)	6		70% (42)	65% (40)	82
		7	7	14		60	62	122
OR 1.642, 95%CI 0.17-16.20, p-value interaction 0.671								
**Westen-dorp et al**	mRS 0-2	80% (557)	76% (535)	1092		39% (216)	40% (220)	436
	mRS 3-6	20% (140)	24% (172)	312		61% (344)	60% (330)	674
		697	707	1404		560	550	1110
OR p-value interaction 1.394 95%CI 0.97-2.01, p-value interaction 0.074								
**Harms et al**	mRS 0-2	-	-	-		40% (12)	27% (8)	20
	mRS 3-6	-	-	-		60% (18)	73% (22)	40
		-	-	-		30	30	60
OR p-value interaction NA								

Post-hoc sample size analysis

A provisional straightforward post hoc power analysis - taking into account the ordinal nature of the modified Rankin Scale, but ignoring clustering by trial and other covariates - showed that observed total sample sizes of 2002 patients in the antibiotics groups and 1999 patients in the control groups of the meta-analysis had 80% power at a 5% two-sided significance level using a Wilcoxon (Mann-Whitney) rank sum test to detect a Cliff's delta =0.05 or higher, the difference in probability of a patient from one group having a better outcome (lower mRS score) than a patient from the other group. This detection margin of 0.05 should be considered as negligible (R-software, package 'effsize' 0.8.1, date 2020-10-05 by M Torchiano), suggesting sufficient power for a clinically more relevant contrast between groups.

Even for a subgroup analysis of placebo controlled trials with only sample sizes of 79 patients in the antibiotics groups and 77 patients in the control groups a similar provisional post hoc power analysis suggested 80% power at a 5% two-sided significance level using a Wilcoxon (Mann-Whitney) rank sum test to detect a Cliff's delta =0.252 or higher. This detection margin should be considered as a small effect size, suggesting sufficient power for a clinically relevant contrast between groups.
